# Supplementary material for: Ischemia‐Induced Post‐Translational Modifications of GLT‐1 Mediate Aberrant Trafficking and Impaired Glutamate Uptake
Source: J Neurochem. 2026 Jun 16;170(6):e70497. doi: 10.1111/jnc.70497 (PMC13270423; doi:10.1111/jnc.70497)
Supplement: Supplementary file 1 — Figure S1: Cellular composition and glutamate transporter expression in primary glial cultures. (A) Representative confocal immunofluorescence images showing astrocyte marker GFAP and neuronal marker MAP2. (B) Representative confocal immunofluorescence images showing astrocyte marker GFAP and microglial marker IBA1. (C) Representative confocal immunofluorescence images showing astrocyte marker GFAP and glutamate transporter GLAST. (D) Representative confocal immunofluorescence images showing astrocyte marker GFAP and glutamate transporter GLT‐1. For all panels, merged image is shown alongside individual channels. Scale bar: 100 μm (E) Quantification of glutamate uptake in presence of 1 μM of the selective GLAST inhibitor UCPH‐101, the selective GLAST/GLT‐1 inhibitor TFB‐TBOA, and the selective GLT‐1 inhibitor WAY 213613. Data are expressed as % of L‐3H‐glutamate normalized to vehicle. Figure S2: Increasing OGD duration reduces GLT‐1 surface expression and transport velocity in primary glial cultures. (A) Representative immunoblots showing GLT‐1 surface and total expression (~65 kDa) following 30 min, 1 h, or 2 h OGD. (B) Quantification of surface (left) and total (right) GLT‐1 expression across varying OGD insult lengths (n = 5–6 independent cell culture preparations per group). (C) Representative Michaelis–Menten saturation curves of L‐3H‐glutamate uptake measured at varying OGD lengths (right) and quantification of V max values normalized to nmol/μg/min (n = 7 independent cell culture preparations per group; 11 replicates averaged per experiment). (D) Representative immunoblots showing GLAST surface and total expression (~65 kDa) following 30 min, 1 or 2 h OGD. (D) Quantification of surface (left) and total (right) GLAST expression across varying OGD insult lengths (n = 5–6 independent cell culture preparations per group). Data are presented as mean ± SEM. Statistical analyses were performed using one way ANOVA followed by Dunnett's multiple comparison post hoc te [file JNC-170-e70497-s001.docx]

**Supplemental material for:**

**Ischemia-Induced Post-Translational Modifications of GLT-1 Mediate Aberrant Trafficking and Impaired Glutamate Uptake**

Authors: Simran Kaur Gill ^1^, Max Kroll ^1^, Katelyn Louise Reeb ^1^, Ole V. Mortensen ^1^, Andréia Cristina Karklin Fontana ^1^*

**Authors affiliation:**

**1-** Department of Pharmacology and Physiology, Drexel University College of Medicine, Philadelphia, PA 19102, USA

**Corresponding author:**

*Andréia C. K. Fontana

Department of Pharmacology and Physiology, Drexel University College of Medicine, Philadelphia, PA, 19102, United States

ORCID: 0000-0002-4791-8746

Tel: +1 215-762-4399. E-mail: acm83@drexel.edu


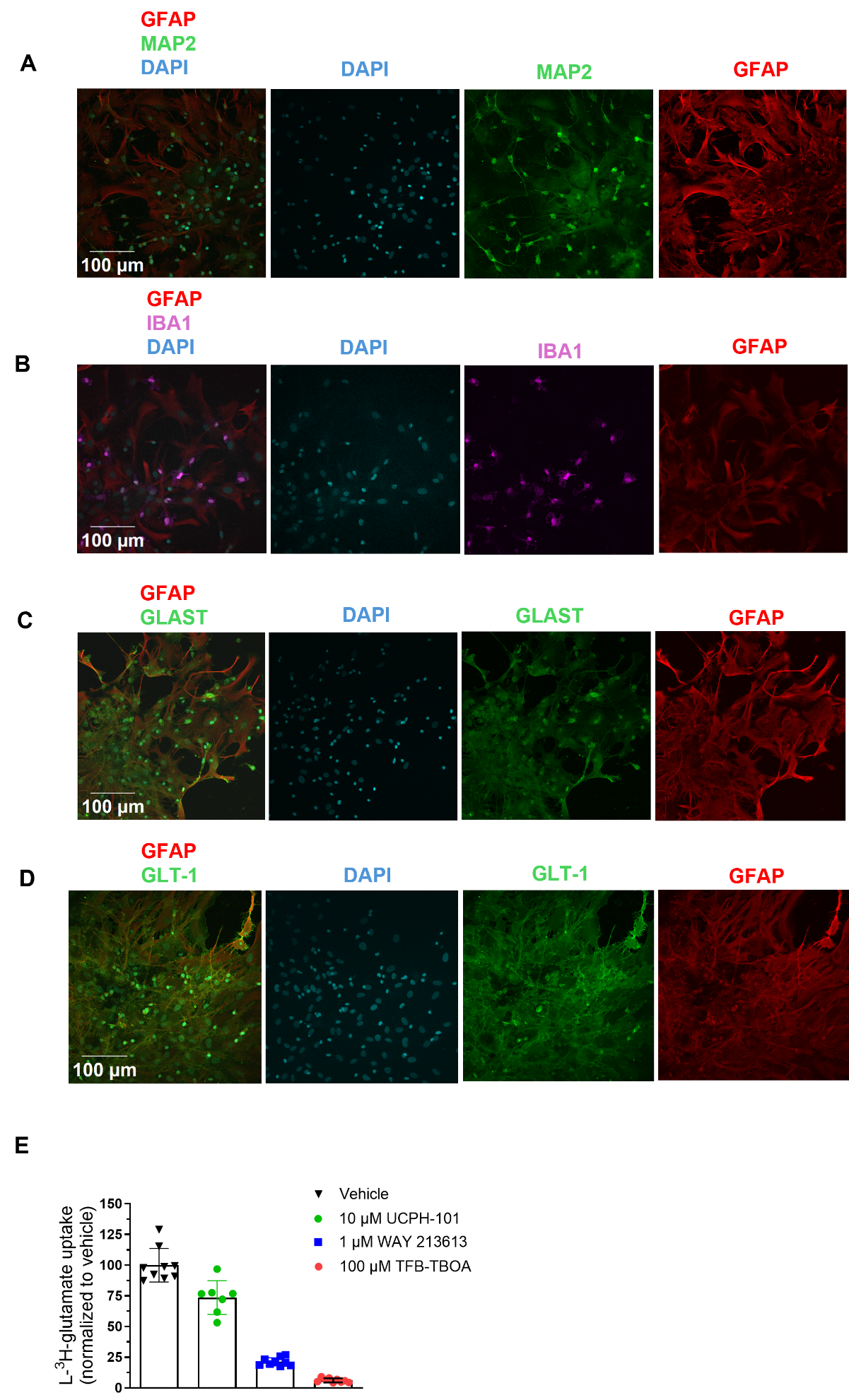


**Supplemental Figure 1. Cellular composition and glutamate transporter expression in primary glial cultures.**

**A**. Representative confocal immunofluorescence images showing astrocyte marker GFAP and neuronal marker MAP2. **B**. Representative confocal immunofluorescence images showing astrocyte marker GFAP and microglial marker IBA1. **C**. Representative confocal immunofluorescence images showing astrocyte marker GFAP and glutamate transporter GLAST. **D**. Representative confocal immunofluorescence images showing astrocyte marker GFAP and glutamate transporter GLT-1. For all panels, merged image is shown alongside individual channels. Scale bar: 100 µm **E.** Quantification of glutamate uptake in presence of 1 µM of the selective GLAST inhibitor UCPH-101, the selective GLAST/GLT-1 inhibitor TFB-TBOA, and the selective GLT-1 inhibitor WAY 213613. Data are expressed as % of L-^3^H-glutamate normalized to vehicle.


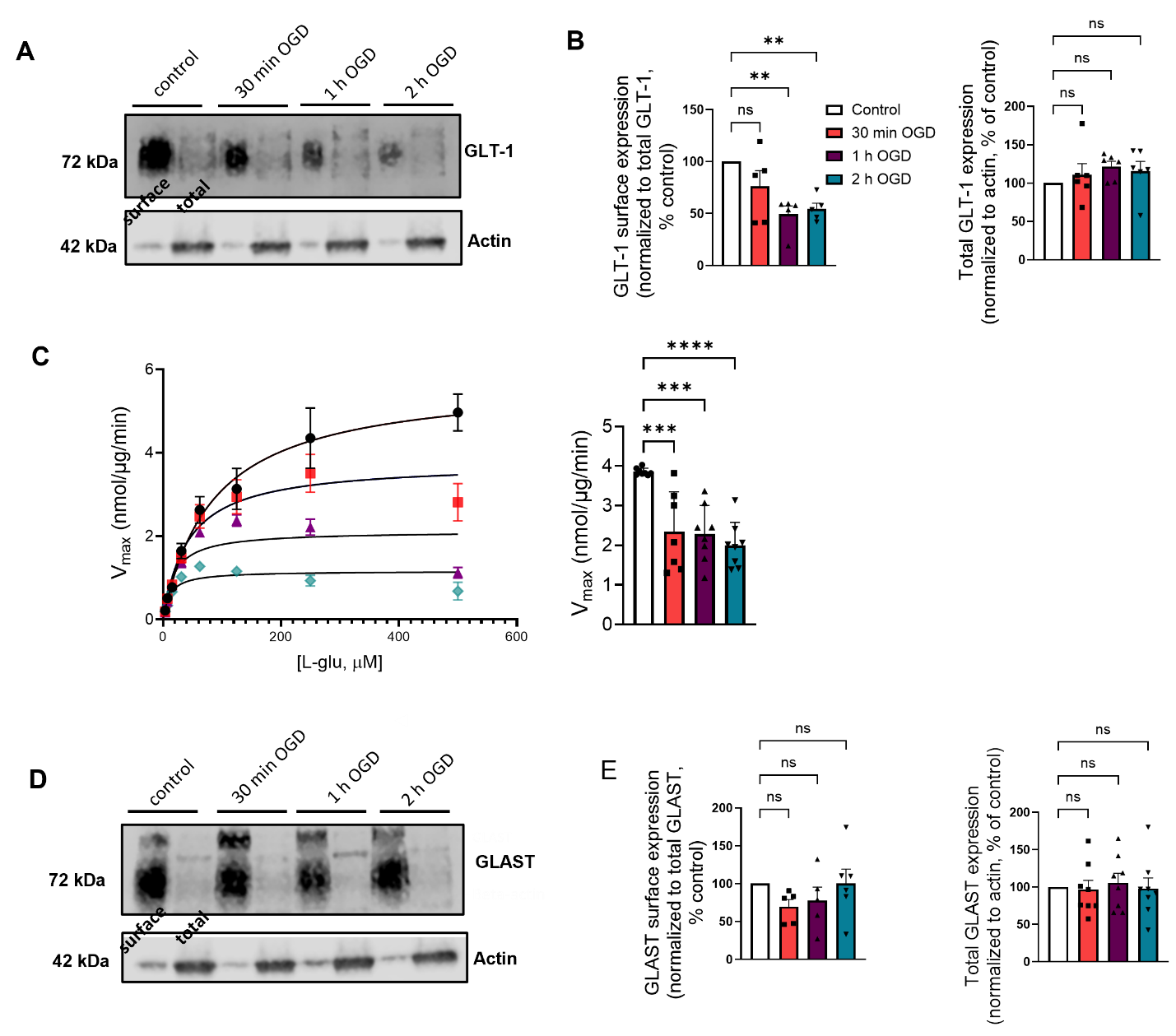


**Supplemental Figure 2. Increasing OGD duration reduces GLT-1 surface expression and transport velocity in primary glial cultures.**

**A.** Representative immunoblots showing GLT-1 surface and total expression (~65 kDa) following 30 minutes, 1 h, or 2 h OGD. **B.** Quantification of surface (left) and total (right) GLT-1 expression across varying OGD insult lengths (n=5-6 independent cell culture preparations per group). **C.** Representative Michaelis-Menten saturation curves of L-^3^H-glutamate uptake measured at varying OGD lengths (right) and quantification of V_max_ values normalized to nmol/μg/min (n=7 independent cell culture preparations per group; 11 replicates averaged per experiment). **D**. Representative immunoblots showing GLAST surface and total expression (~65 kDa) following 30 minutes, 1 h, or 2 h OGD. **D.** Quantification of surface (left) and total (right) GLAST expression across varying OGD insult lengths (n=5-6 independent cell culture preparations per group). Data are presented as mean ± SEM. Statistical analyses were performed using One Way ANOVA followed by Dunnett’s multiple comparison *post-hoc* test. ** *p* < 0.01, *** *p* < 0.001, **** *p* < 0.00001, ns= non-significant.


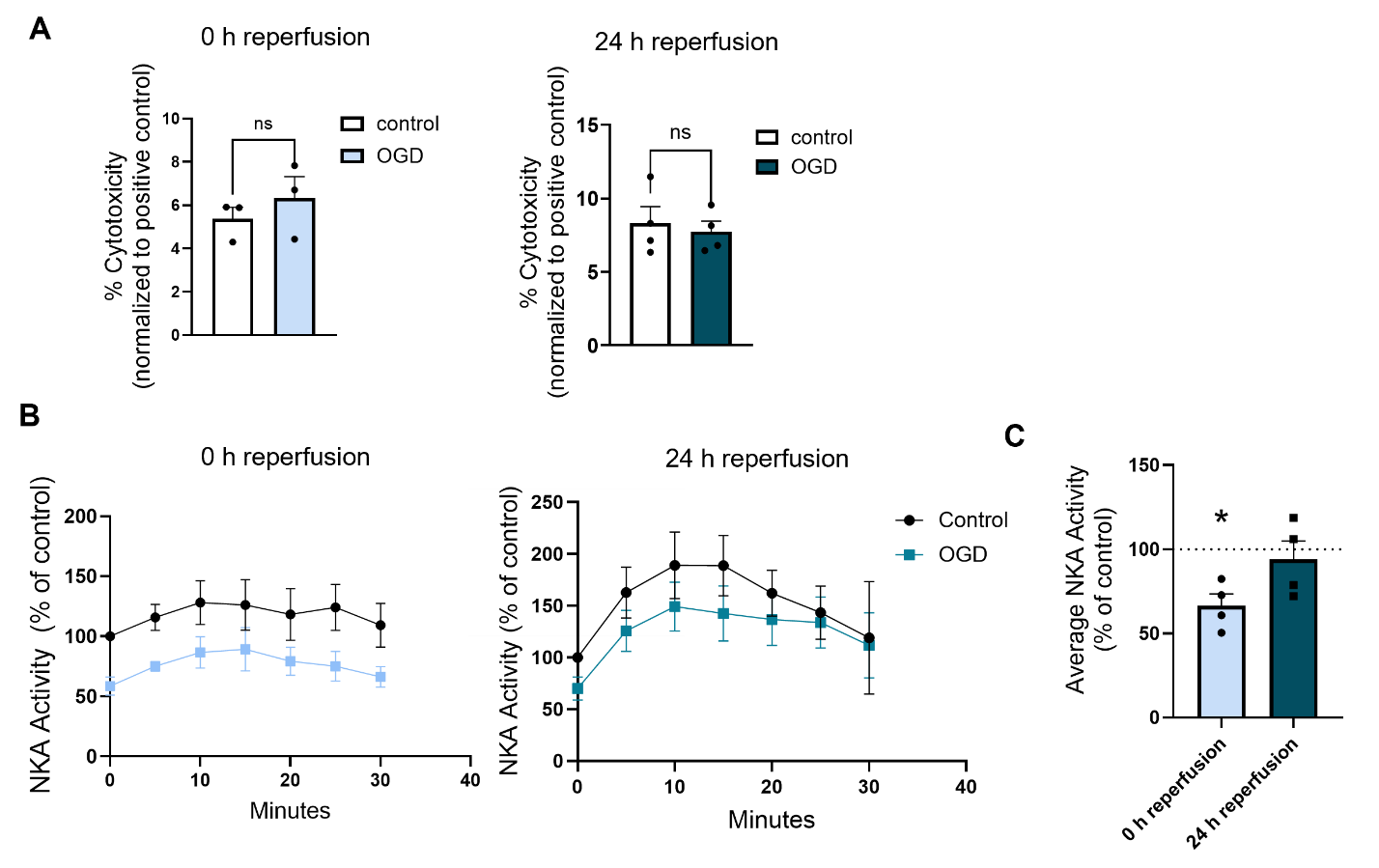


**Supplemental Figure 3. OGD does not induce cytotoxicity in glia cultures and transiently reduces Na⁺/K⁺-ATPase activity.**

**A.** Lactate dehydrogenase assay measuring level of cytotoxicity in glia cultures following OGD (n= 4 from independent cell cultures per group with six replicates averaged) **B.** Enzymatic activity of the NKA measured through a phosphatase fluorometric assay over a period of 35 min immediately following OGD (left) or after 24-hour reperfusion (right) (n= 5 from independent cell cultures per group with duplicates averaged). **C**. Average enzymatic activity of NKA at 4 h and 24 h reperfusion measured throughout 35 min reading, expressed as a percentage of the control. Data is presented as mean ± SEM. Statistical analyses were performed using One Way ANOVA followed by Dunnett’s multiple comparison *post-hoc* test. *** *p* < 0.001, ns= non-significant.


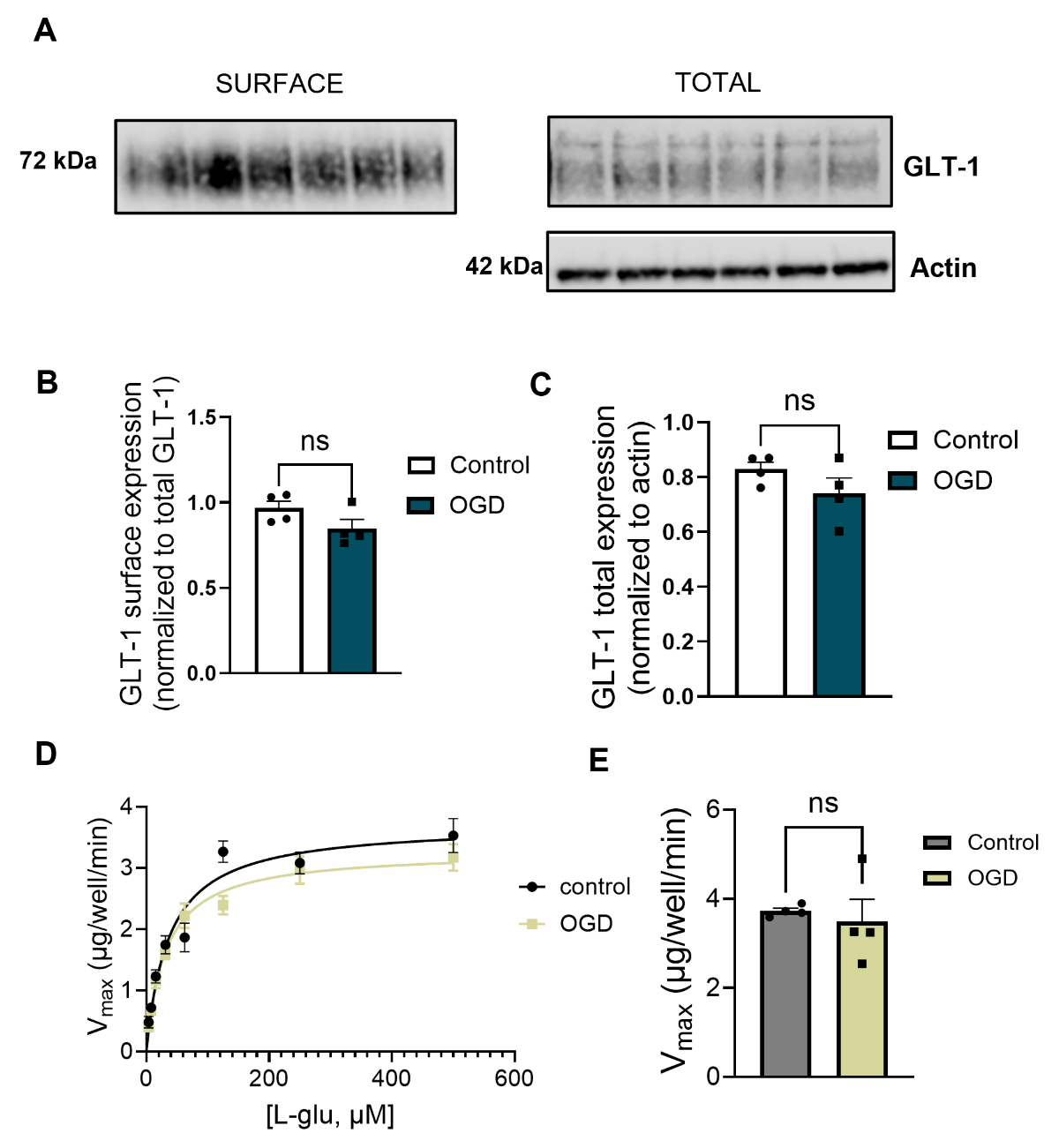


**Supplemental Figure 4. GLT-1 surface expression and glutamate transport velocity recover by 48 h reperfusion following OGD in primary glial cultures.**

**A.** Representative immunoblots showing GLT-1 surface and total expression (~65 kDa) following 2 h OGD and 48 h reperfusion. **B-C.** Quantification of surface (**B**) and total (**C**) GLT-1 expression following 2 h OGD and 48 h reperfusion (n=4 independent cell culture preparations per group; triplicates wells averaged). **D**. Representative Michaelis-Menten saturation curves of L-^3^H-glutamate uptake measured at 48 h reperfusion. **E**. Quantification of V_max_ values normalized to nmol/μg/min following 48 reperfusion (n=4 independent cell culture preparations per group; 11 technical replicates averaged per experiment). Data are presented as mean ± SEM. Statistical analyses were performed using two-tailed unpaired t tests. ns= non-significant.


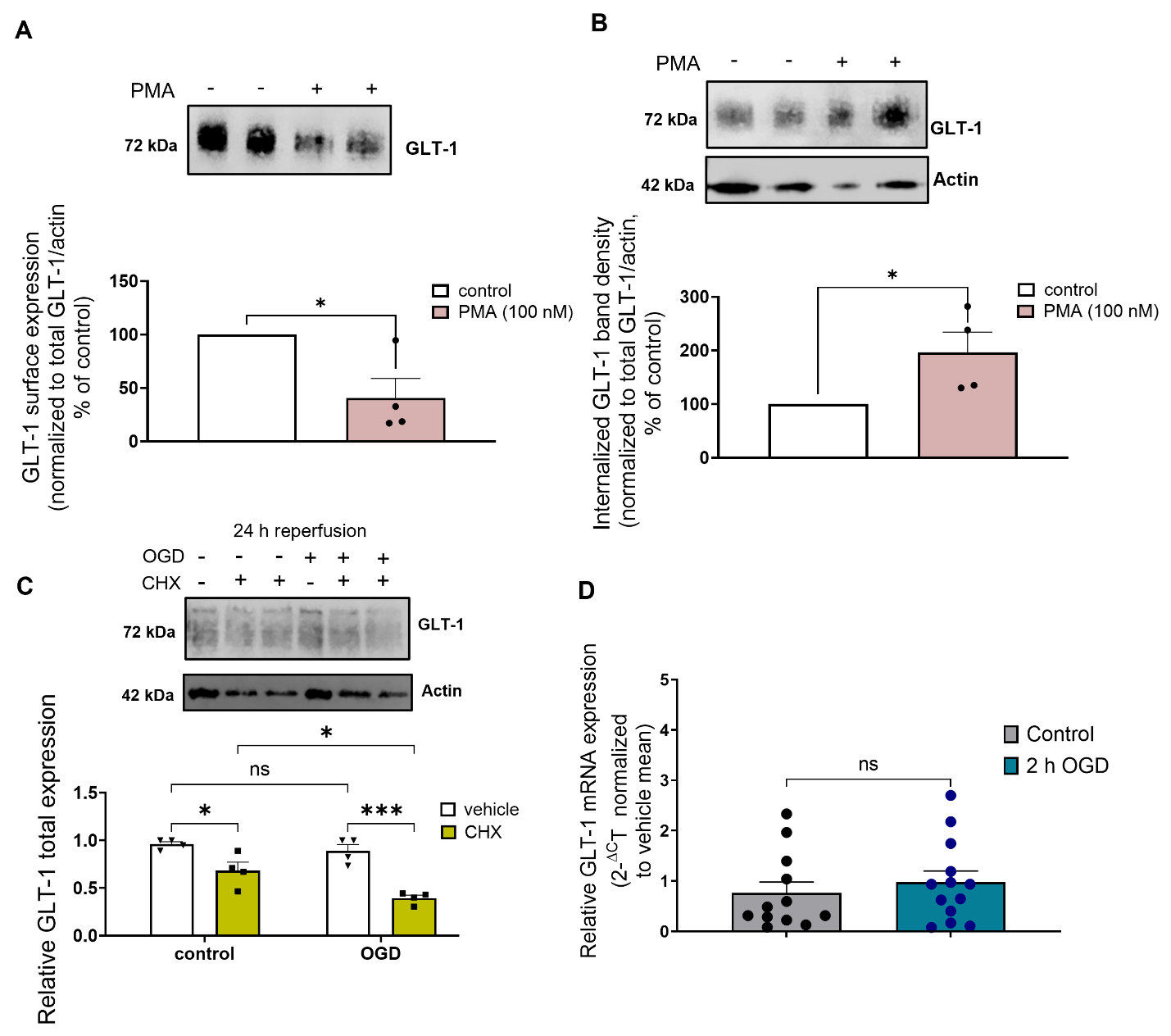


**Supplemental Figure 5. GLT-1 internalization, protein turnover, and transcriptional regulation in primary glial cultures.**

**A.** Representative immunoblots (top) and protein quantification (bottom) of surface expressed GLT-1 following treatment with 100 nM PMA. **B**. Representative immunoblot (top) and protein quantification (bottom) of internalized pre-labeled GLT-1 measured by endocytic biotinylation following treatment with 100 nM PMA (n=4 independent cell culture preparations per group; technical duplicates averaged). **C**. Representative immunoblot (top) and protein quantification (bottom) of total GLT-1 expression following 24 h reperfusion with cycloheximide (CHX, 25mM) applied immediately after OGD. **D**. Relative GLT-1 mRNA expression levels measured by qPCR analysis, normalized to housekeeping gene expression, displayed at 2-^ΔCT^ (n=13). Data are presented as mean ± SEM. Statistical analyses were performed using a two-tailed unpaired t test or Two Way ANOVA followed by Šidák’s *post-hoc* test. * *p* < 0.05, ** *p* < 0.01, ns = non-significant.


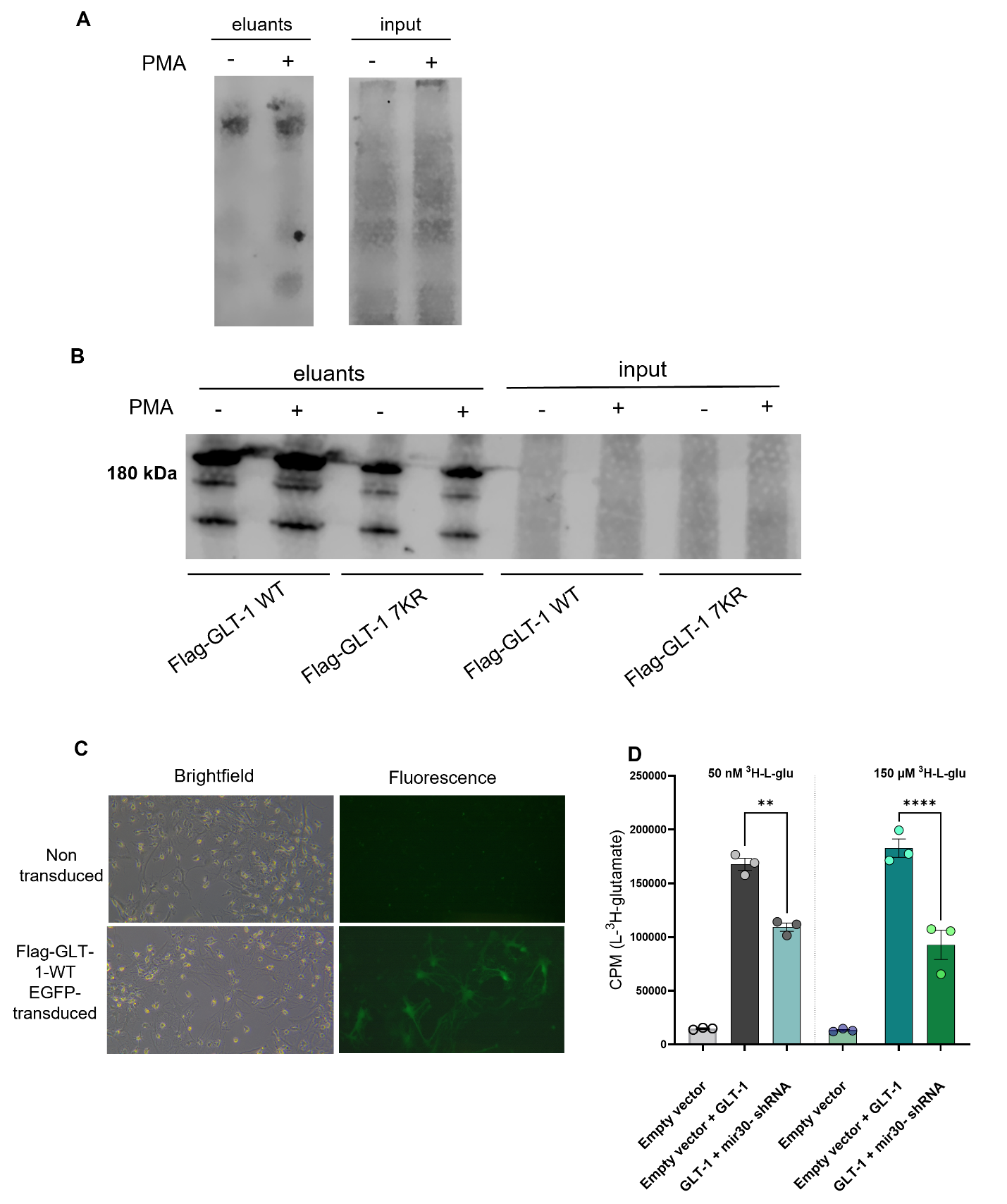


**Supplemental Figure 6. Validation of GLT-1 immunoprecipitation, viral constructs, and miR30 knockdown efficiency.**

**A.** PMA treatment increased ubiquitinated GLT-1 in primary glial cultures, serving as a positive control for the immunoprecipitation approach used in Figure 4. GLT-1 was immunoprecipitated from glial cultures treated with vehicle or PMA and immunoblotted for ubiquitin. **B**. COS-7 cells transfected with Flag-tagged GLT-1 (WT or 7KR) were treated with PMA for 90 min followed by Flag immunoprecipitation and immunoblotting for ubiquitin (P4D1) **C**. Representative immunofluorescence images of glia cultures transduced with Flag-GLT-1 WT, compared to non-transduced cells, visualized using a GFP filter cube to detect EGFP fluorescence. **D**. Quantification L-^3^H-glutamate uptake measured in COS-7 cells after transfection with empty vector, empty vector + GLT-1, or GLT-1 + mir30 siRNA, demonstrating knockdown efficiency of GLT-1. Data are presented as mean ± SEM. Statistical analyses were performed using a One Way ANOVA followed by Šídák’s multiple comparison *post-hoc* test. ** *p* < 0.01, **** *p* < 0.0001.

**
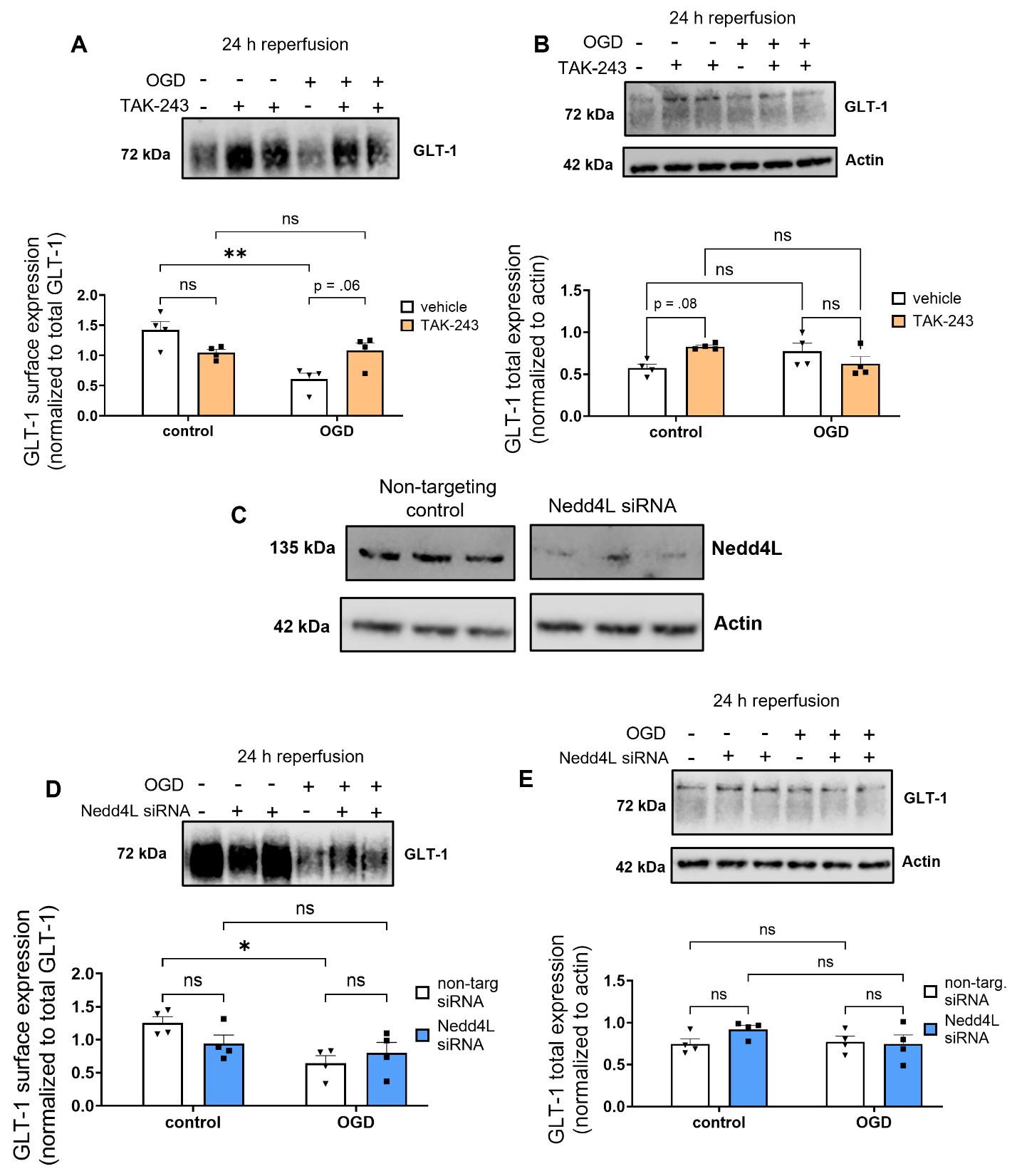
**

**Supplemental Figure 7. Inhibition of GLT-1 ubiquitination prevents OGD-induced surface downregulation in primary glial cultures.**

**A-B**. Representative immunoblots (top), and protein quantification (bottom) of surface (**A**) and total (**B**) GLT-1 expression following 24 h reperfusion with TAK-243 (100 nM) applied immediately after OGD. **C.** Representative immunoblot showing Nedd4L expression 72 h after transfection with Nedd4L siRNA or non-targeting control. **D-E**. Glial cultures were transfected with Nedd4L siRNA 72 h prior to OGD. Representative immunoblots (top), and protein quantification (bottom) of surface (**D**) and (**E**) GLT-1 expression at 24 h reperfusion. Data (**A-B, D-E**) are presented as mean ± SEM (n=4 independent culture preparations per group). Statistical analyses were performed using Two Way ANOVA followed by Šidák’s *post-hoc* test. ** *p* < 0.01, *** *p* < 0.001, ns= non-significant.


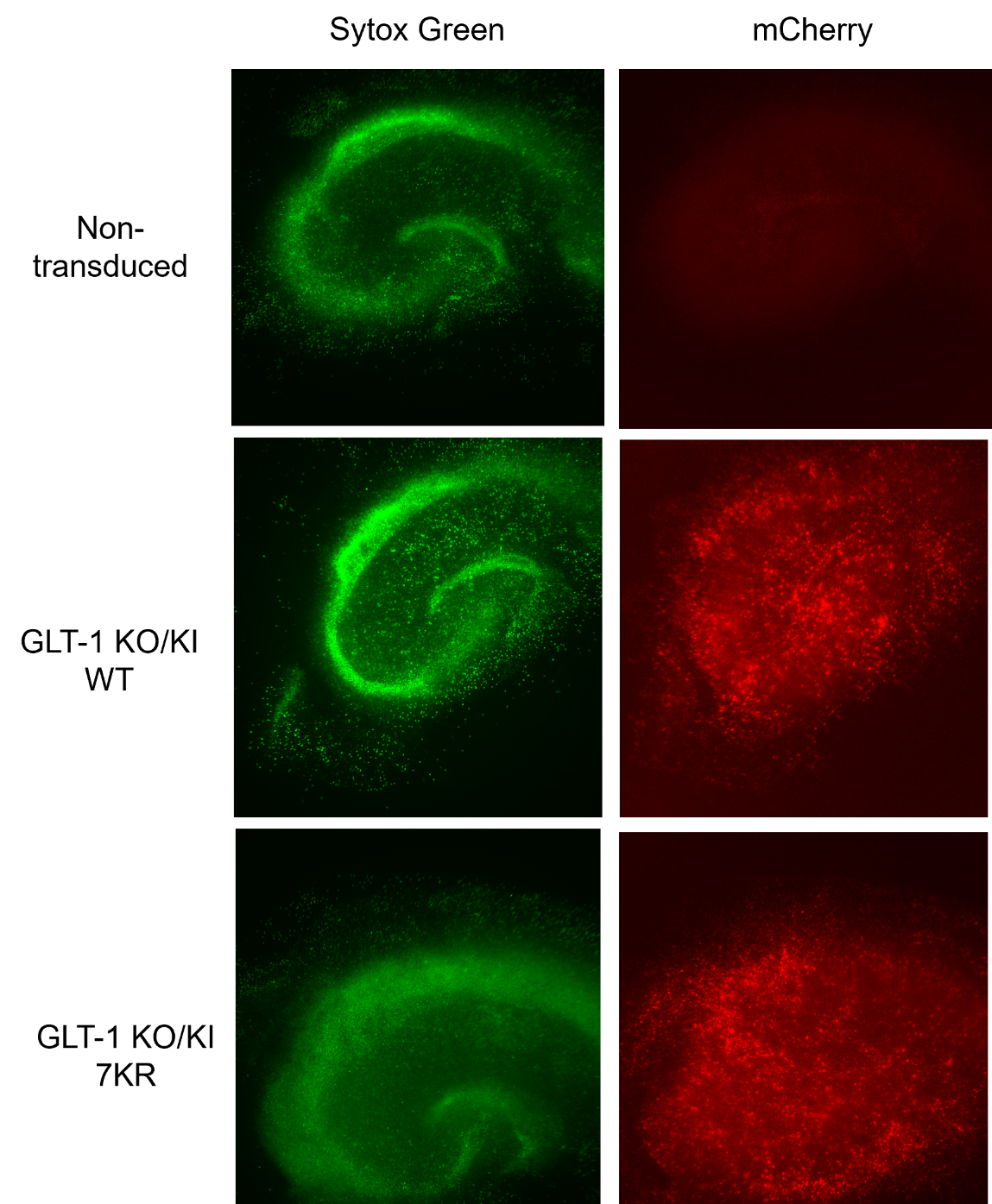


**Supplemental Figure 8. Validation of lentiviral transduction in hippocampal organotypic slice cultures (OSCs).** Representative immunofluorescence images of hippocampal OCSs transduced with GLT-1 KO/KI WT, compared to non-transduced slices, following OGD and Sytox Green staining. mCherry signal (red fluorescence) indicates successful viral transduction within the slice cultures.

**Supplemental Table 1: Normality Test for All Figures**

| **Figure** | | **Shapiro-Wilk test for Normality** |
| --- | --- | --- |
| 1 | E | Control: W = 0.8978, *p* = 0.1488  0 h RF: W = 0.9061, *p* = 0.4621  4 h RF: W = 0.9141, *p* = 0.5045  24 h: W = 0.8515, *p* = 0.2320 |
|  | G | Control: W = 0.9212, *p* = 0.0706  0 h RF: W = 0.8476, *p* = 0.2185  1 h RF: W = 0.9457, *p* =0.6896  2 h RF: W = 0.8517, *p* = 0.2319  4 h RF: W = 0.9230, *p* = 0.5541  6 h RF: W = 0.7813, *p* = 0.0705  24 h RF: W = 0.9504, *p* = 0.7183 |
|  | H | Control: W = 0.9860, *p* = 0.9797  0 h RF: W = 0.8694, *p* = 0.2953  1 h RF: W = 0.9824, *p* = 0.9160  2 h RF: W = 0.9366, *p* = 0.6337  4 h RF: W = 0.9766, *p* = 0.8819  6 h RF: W = 0.9832, *p* = 0.7516  24 h RF: W = 0.8751, *p* = 0.3181 |
| 2 | B | Control: W= 0.8883, *p* = 0.3752  OGD: W = 0.9293, *p* = 0.5906 |
|  | C | Control: W = 0.9534, *p* = 0.7615  OGD: W = 0.9095, *p* = 0.4647 |
|  | D | Control: W = 0.8407, *p* = 0.1975  OGD: W = 0.8236, *p* = 0.1518 |
|  | E | Control: W = 0.9577, *p* = 0.7916  OGD: W = 0.9034, *p* = 0.4290 |
|  | F | Control vehicle: W = 0.7730, *p* = 0.0620  Control Dynasore: W = 0.9600, *p* = 0.7788  OGD vehicle: W = 0.9222, *p* = 0.5494  OGD Dynasore: W = 0.9717, *p* = 0.8518 |
|  | G | Control vehicle: W = 0.8558, *p* = 0.2456  Control Dynasore: W = 0.9132, *p* = 0.4997  OGD vehicle: W = 0.8607, *p* = 0.2629  OGD Dynasore: W = 0.9072, *p* = 0.4678 |
|  | I | Control: W = 0.7891, *p* = 0.0841  OGD: W = 0.8527, *p* = 0.2352 |
|  | J | Control: W = 0.9307, *p* = 0.5986  OGD: W = 0.9602, *p* = 0.7801 |
| 3 | B | Control vehicle: W = 0.8739, *p* = 0.3133  Control MG-132: W = 0.9604, *p* = 0.7817  OGD vehicle: W = 0.8021, *p* = 0.1060  OGD MG-132: W = 0.9992, *p* = 0.9979 |
|  | C | Control vehicle: W = 0.9754, *p* = 0.8748  Control MG-132: W = 0.7834, *p* = 0.0756  OGD vehicle: W = 0.9147, *p* = 0.5074  OGD MG-132: W = 0.8516, *p* = 0.2314 |
|  | D | Control vehicle: W = 0.7973, *p* = 0.1081  Control BAF: W = 0.9123, *p* = 0.4947  OGD vehicle: W = 0.8715, *p* = 0.3037  OGD BAF: W = 0.8688, *p* = 0.2932 |
|  | E | Control vehicle: W = 0.8845, *p* = 0.3580  Control BAF: W = 0.9004, *p* = 0.4332  OGD vehicle: W = 0.9904, *p* = 0.9596  OGD BAF: W = 0.8314, *p* = 0.1714 |
|  | F | Control vehicle: W = 0.9933, *p* = 0.9735  Control BAF + MG-132: W = 0.9906, *p* = 0.9606  OGD vehicle: W = 0.9138, *p* = 0.5028  OGD BAF + MG-132: W = 0.7682, *p* = 0.0563 |
|  | G | Control vehicle: W = 0.8990, *p* = 0.4261  Control BAF + MG: W = 0.7889, *p* = 0.0837  OGD vehicle: W = 0.8304, *p* = 0.1688  OGD BAF + MG-132: W = 0.7694, *p* = 0.0577 |
| 4 | A | Control: W = 0.9763, *p* = 0.8798  OGD: W = 0.9302, *p* = 0.5956 |
|  | B | Control: W = 0.9455, *p* = 0.6882  OGD: W = 0.9927, *p* = 0.9707 |
|  | C | Control: W = 0.8474, *p* = 0.2335  OGD: W = 0.8729, *p* = 0.3038 |
|  | D | Control: W = 0.9310, *p* = 0.4922  OGD: W = 0.4922, *p* = 0.9438 |
| 5 | B | Control WT: W = 0.9235, *p* = 0.5566  Control 7KR: W = 0.9087, *p* = 0.4754  OGD WT: W = 0.8330, *p* = 0.1757  OGD 7KR: W = 0.9520, *p* = 0.7284 |
|  | C | Control WT: W = 0.8645, *p* = 0.2765  Control 7KR: W = 0.8836, *p* = 0.3543  OGD WT: W = 0.8479, *p* = 0.2194  OGD 7KR: W = 0.8787, *p* = 0.3330 |
|  | D | Control WT: W = 0.9181, *p* = 0.5261  Control 7KR: W = 0.9523, *p* = 0.7306  OGD WT: W = 0.9880, *p* = 0.9470  OGD 7KR: W = 0.9053, *p* = 0.4576 |
|  | E | Control WT: W = 0.8519, *p* = 0.2323  Control 7KR: W = 0.9894, *p* = 0.9545  OGD WT: W = 0.9226, *p* = 0.5517  OGD 7KR: W = 0.8837, *p* = 0.3546 |
| 6 | B | Control WT: W = 0.8301, *p* = 0.1680  Control 7KR: W = 0.8429, *p* = 0.2041  OGD WT: W = 0.7776, *p* = 0.0677  OGD 7KR: W = 0.9213, *p* = 0.5441 |
|  | D | Control WT: W = 0.8708, *p* = 0.3010  Control 7KR: W = 0.9681, *p* = 0.8296  OGD WT: W = 0.8710, *p* = 0.2983  OGD 7KR: W = 0.9276, *p* = 0.5804 |
|  | F | Control WT: W = 0.9456, *p* = 0.6889  Control 7KR: W = 0.9061, *p* = 0.4619  OGD WT: W = 0.9157, *p* = 0.5130  OGD 7KR: W = 0.9569, *p* = 0.7593 |
| 7 | B | Control vehicle: W = 0.8186, *p* = 0.1399  Control TAK-243: W = 0.9254, *p* = 0.5676  OGD vehicle: W = 0.9605, *p* = 0.7821  OGD TAK-243: W = 0.8183, *p* = 0.1392 |
|  | C | Control vehicle: W = 0.9373, *p* = 0.6380  Control TAK-243: W = 0.8686, *p* = 0.2923  OGD vehicle: W = 0.9175, *p* = 0.5230  OGD TAK-243: W = 0.9081, *p* = 0.4723 |
|  | D | Control non-targ: W = 0.9811, *p* = 0.9087  Control Nedd4L: W = 0.9540, *p* = 0.7414  OGD non-targ: W = 0.8635, *p* = 0.2729  OGD Nedd4L: W = 0.9245, *p* = 0.5626 |
|  | E | Control non-targ: W = 0.9194, *p* = 0.5336  Control Nedd4L: W = 0.9562, *p* = 0.7548  OGD non-targ: W = 0.8285, *p* = 0.1845  OGD Nedd4L: W = 0.9846, *p* = 0.9287 |
| 8 | B | Control: W = 0.9512, *p* = 0.7239  OGD: W = 0.9003, *p* = 0.4326 |
|  | C | Control: W = 0.8197, *p* = 0.1425  OGD: W = 0.9910, *p* = 0.9623 |
|  | D | Control: W = 0.8948, *p* = 0.4059  OGD: W = 0.7909, *p* = 0.0868 |
|  | E | Control: W = 0.9977, *p* = 0.9924  OGD: W = 0.7731, *p* = 0.0518 |
|  | G | CA1  OGD WT: W = 0.9074, *p* = 0.4523, OGD 7KR: W = 0.9076, *p* = 0.4535, MK801: W = 0.9413, *p* = 0.6620  CA3  OGD WT: W = 0.9121, *p* = 0.4803, OGD 7KR: W = 0.8222, *p* = 0.1214, MK801: W = 0.9722, *p* = 0.8553  Dentate Gyrus  OGD WT: W = 0.9121, *p* = 0.4803, OGD 7KR: W = 0.8222, *p* = 0.1214, MK801: W = 0.9722, *p* = 0.8553 |
| S1 | B | Vehicle: W = 0.8593, *p* =0.1183  10 μM UCPH-101: W= 0.6357, *p* = 0.9351  1 μM WAY 213613: W=0.8947, *p* = 0.2231  100 μM TFB-TBOA: W= 0.9397, *p* =0.5310 |
| S2 | B | Surface  30 min OGD: W = 0.8843, *p* = 0.3291  1 h OGD: W = 0.8609, *p* = 0.2634  2 h OGD: W = 0.9513, *p* = 0.7465  Total  30 min OGD: W = 0.8533, *p* = 0.1672  1 h OGD: W = 0.8176, *p* = 0.0841  2 h OGD W = 0.8092, *p* = 0.0710 |
|  | C | Control: W = 0.8752, *p* = 0.1396  30 min OGD: W = 0.9534, *p* = 0.7609  1 h OGD: W = 0.8711, *p* = 0.1264  2 h OGD: W = 0.9448, *p* = 0.6823 |
|  | E | Surface  30 min OGD: W = 0.8113, *p* = 0.0999  1 h OGD: W = 0.9964, *p* = 0.9966  2 h OGD: W = 0.9479, *p* = 0.7229  Total  30 min OGD: W = 0.8988, *p* = 0.2821  1 h OGD: W = 0.9284, *p* = 0.5017  2 h OGD: W = 0.9096, *p* = 0.3509 |
| S3 | A | Control: W = 0.9075, *p* = 0.4693  24 h RF OGD: W = 0.9250, *p* = 0.5653 |
|  | C | 0 h RF: W = 0.9842, *p* = 0.9261  24 h RF: W = 0.9120, *p* = 0.4933 |
| S4 | B | Control: W = 0.8195, *p* = 0.1420  OGD: W = 0.8252, *p* = 0.1557 |
|  | C | Control: W = 0.8690, *p* = 0.2938  OGD: W = 0.9947, *p* = 0.9800 |
|  | E | Control: W = 0.9585, *p* = 0.7694  OGD: W = 0.8783, *p* = 0.3315 |
| S5 | A | Control: W = 0.8763, *p* = 0.3232  PMA: W = 0.9579, *p* = 0.7657 |
|  | B | Control: W = 0.8925, *p* = 0.3947  PMA: W = 0.9654, *p* = 0.8126 |
|  | C | Control vehicle: W = 0.8609, *p* = 0.2635  Control CHX: W = 0.9458, *p* = .6905  OGD vehicle: W = 0.8518, *p* = 0.2319  OGD CHX: W = 0.7855, *p* = 0.0787 |
|  | D | Control: W = .9657, p = 0.8384  OGD: W = .9370, p = 0.4192 |
| S6 | D | 50 μM  Empty vector: W = 0.9915, *p* = 0.8235  Empty vector + GLT-1: W = 0.9865, *p* = 0.7773  GLT-1 + mir30: W = 0.8854, *p* = 0.3404  100 μM  Empty vector: W = 0.9544, *p* = 0.5889  Empty vector + GLT-1: W = 0.9041, *p* = 0.3987  GLT-1 + mir30: W = 0.7835, *p* = 0.0756 |
| S7 | A | Control vehicle: W = 0.9558, *p* = 0.7524  Control TAK-243: W = 0.9385, *p* = 0.6454  OGD vehicle: W = 0.7669, *p* = 0.0549  OGD TAK-243: W = 0.7743, *p* = 0.0636 |
|  | B | Control vehicle: W = 0.9983, *p* = 0.9946  Control TAK-243: W = 0.8591, *p* = 0.2572  OGD vehicle: W = 0.8653, *p* = 0.2795  OGD TAK-243: W = 0.7891, *p* = 0.0841 |
|  | D | Control non-targ: W = 0.8288, *p* = 0.1648  Control Nedd4L: W = 0.8632, *p* = 0.2717  OGD non-targ: 0.8816, *p* = 0.3456  OGD Nedd4L: W = 0.9380, *p* = 0.6422 |
|  | E | Control non-targ: W = 0.8857, *p* = 0.3636  Control Nedd4L: W = 0.7862, *p* = 0.0797  OGD non-targ: W = 0.9991, *p* = 0.9975  OGD Nedd4L: W = 0.9989, *p* = 0.9967 |

**Supplemental Table 2: Statistical Analysis of the Supplemental Figures**

| **Figure** | | **Statistical Test** | **Adjusted P** | **F-value: F (DFn, DFd) or T-value t(df)** |
| --- | --- | --- | --- | --- |
| S2 | B | One-Way ANOVA  (Dunnett’s multiple comparisons) | 0.0105 (control vs 1 h OGD)  0.0043 (control v 2 h OGD) | F (3 ,15) = 6.100, *p* = 0.0063 |
|  | C |  | 0.0083 (control vs 30 min OGD)  0.0001 (control vs 1 h OGD)  0.0009 (control vs 2 h OGD) | F (3, 28) = 9.510, *p* = 0.0002 |
| S3 | C | One-sample *t* test | 0.0173 (0 h RF)  0.6210 (24 h RF) | 0 h: t (3) = 4.786  24 h: t (3) = 0.6210 |
| S5 | A | Two tailed Unpaired *t* test | 0.0176 | t (6) = 3.244 |
|  | B | Two tailed Unpaired *t* test | 0.0441 | t (6) = 2.540 |
|  | C | 2-Way ANOVA  (Šidák’s *post-hoc*) | 0.0345 (control vs control CHX)  0.0251 (control CHX vs OGD CHX)  0.0004 (OGD vehicle vs OGD CHX) | OGD Effect: F (1, 12) = 6=9.553  *p* = 0.0093  Treatment Effect: F (1, 12) = 43.77  *p* = 0.0001 |
| S6 | C | One-Way ANOVA | <0.0001 | F (5, 12) = 102.9  *p* < 0.0001 |
| S7 | A | 2-Way ANOVA  (Šidák’s *post-hoc*) | 0.0013 (control vs OGD in vehicle group)  0.0615 (vehicle vs TAK in OGD group) | OGD x Treatment: F (1, 12) = 14.84,  *p* = 0.0023  OGD Effect: F (1, 12) = 12.51,  *p* = 0.0041 |
|  | B | 2-Way ANOVA  (Šidák’s *post-hoc*) | 0.0870 (vehicle vs TAK in control group) | OGD x Treatment: F (1, 12) = 8.813,  *p* = 0.0117 |
|  | D | 2-Way ANOVA  (Šidák’s *post-hoc*) | 0.0311 (control vs vehicle in OGD group) | OGD x Treatment Effect: F (1, 12) = 3.433  *p* = 0.0887  OGD Effect: F (1, 12) = 8.744  *p* = 0.0120 |

**Uncropped Blots of All Representative Western Blots**

**
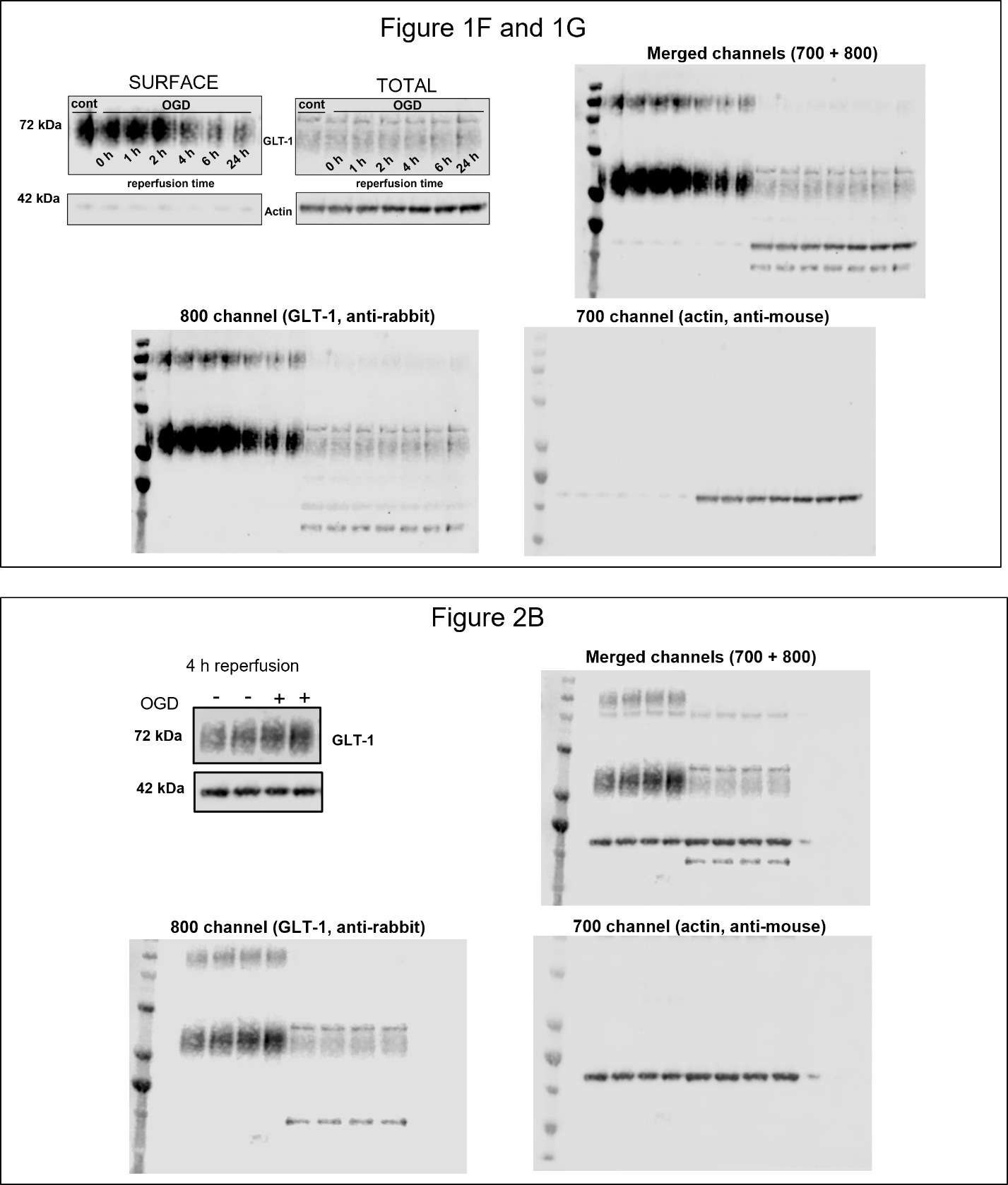
**

**
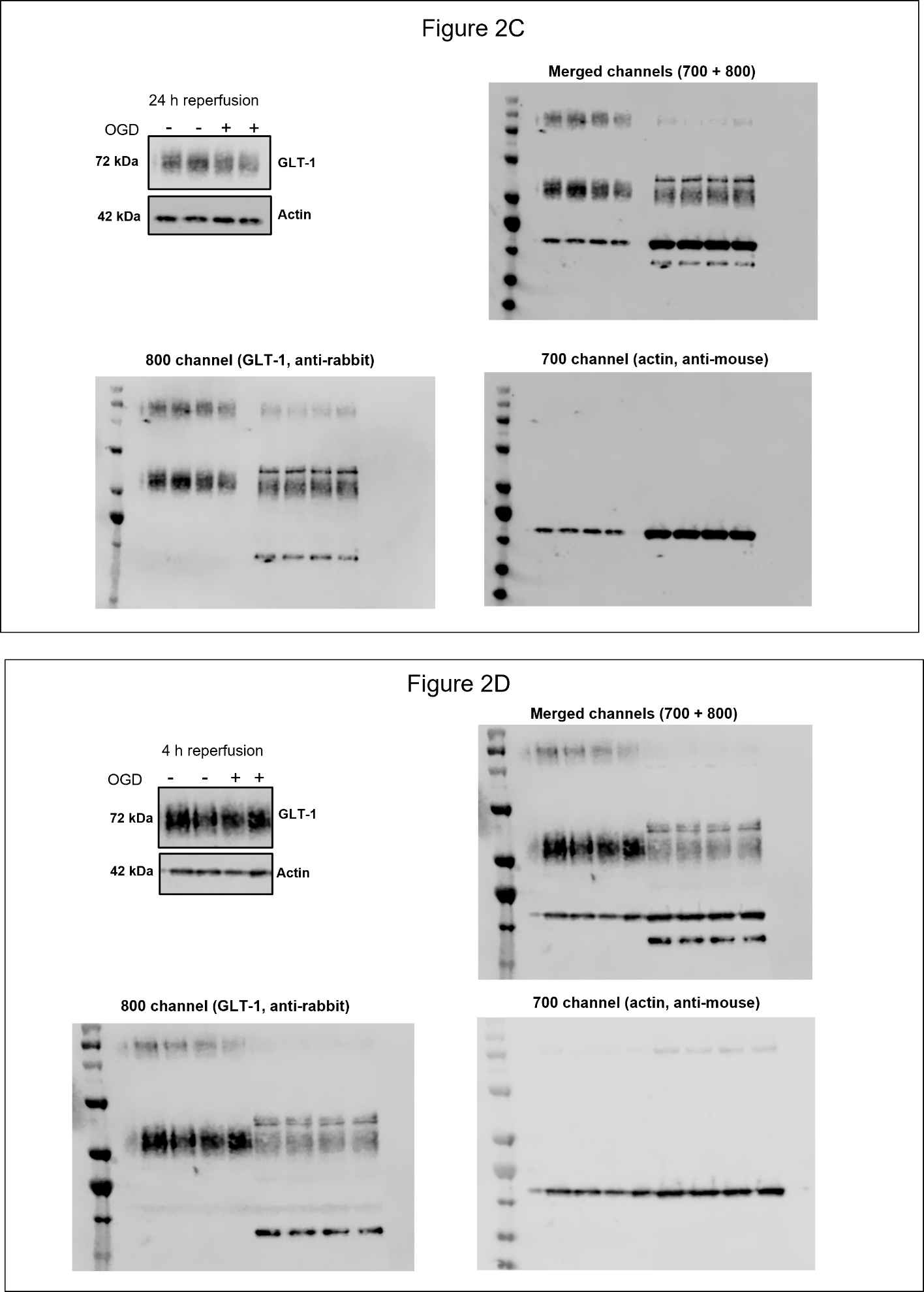

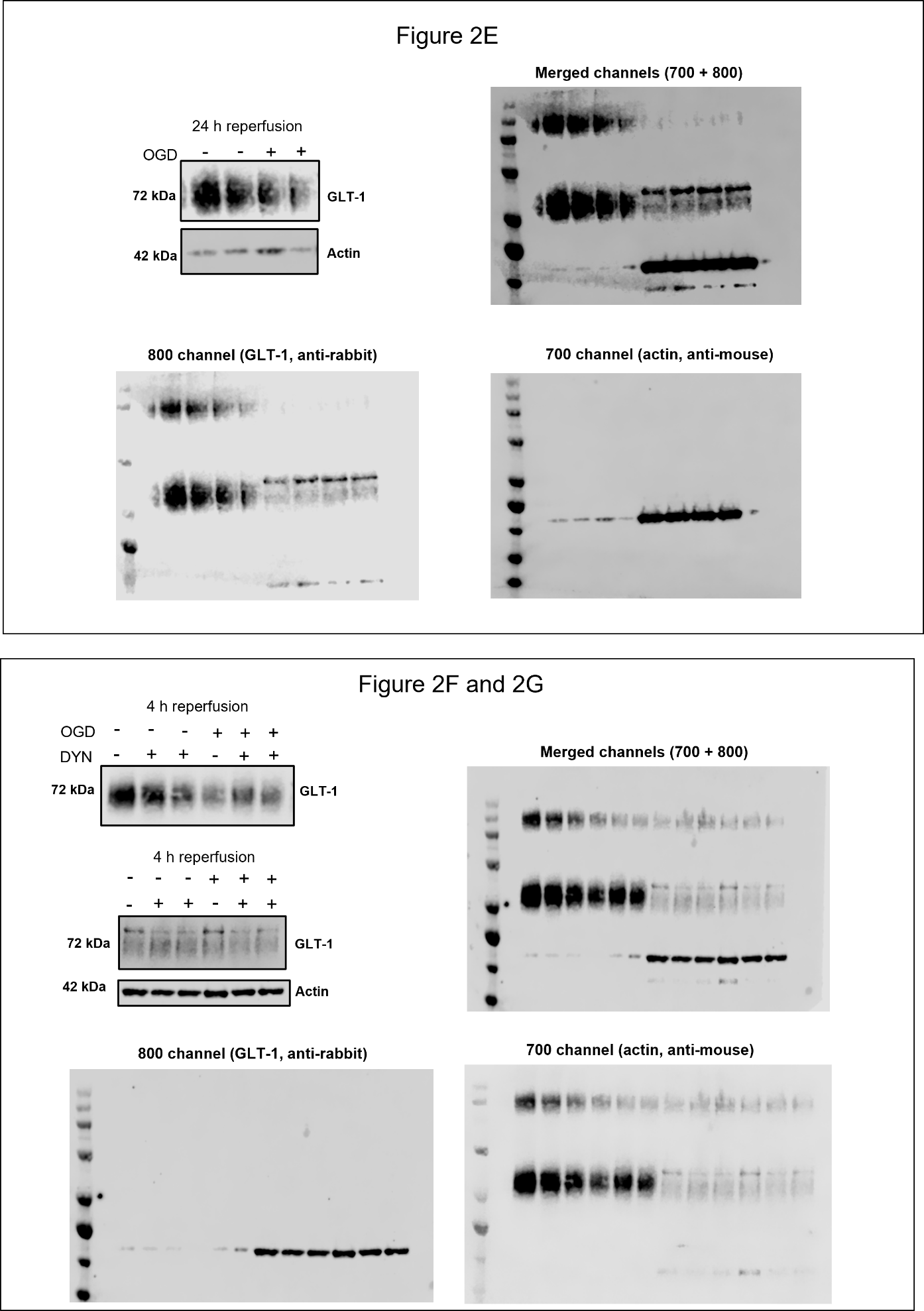
**

**
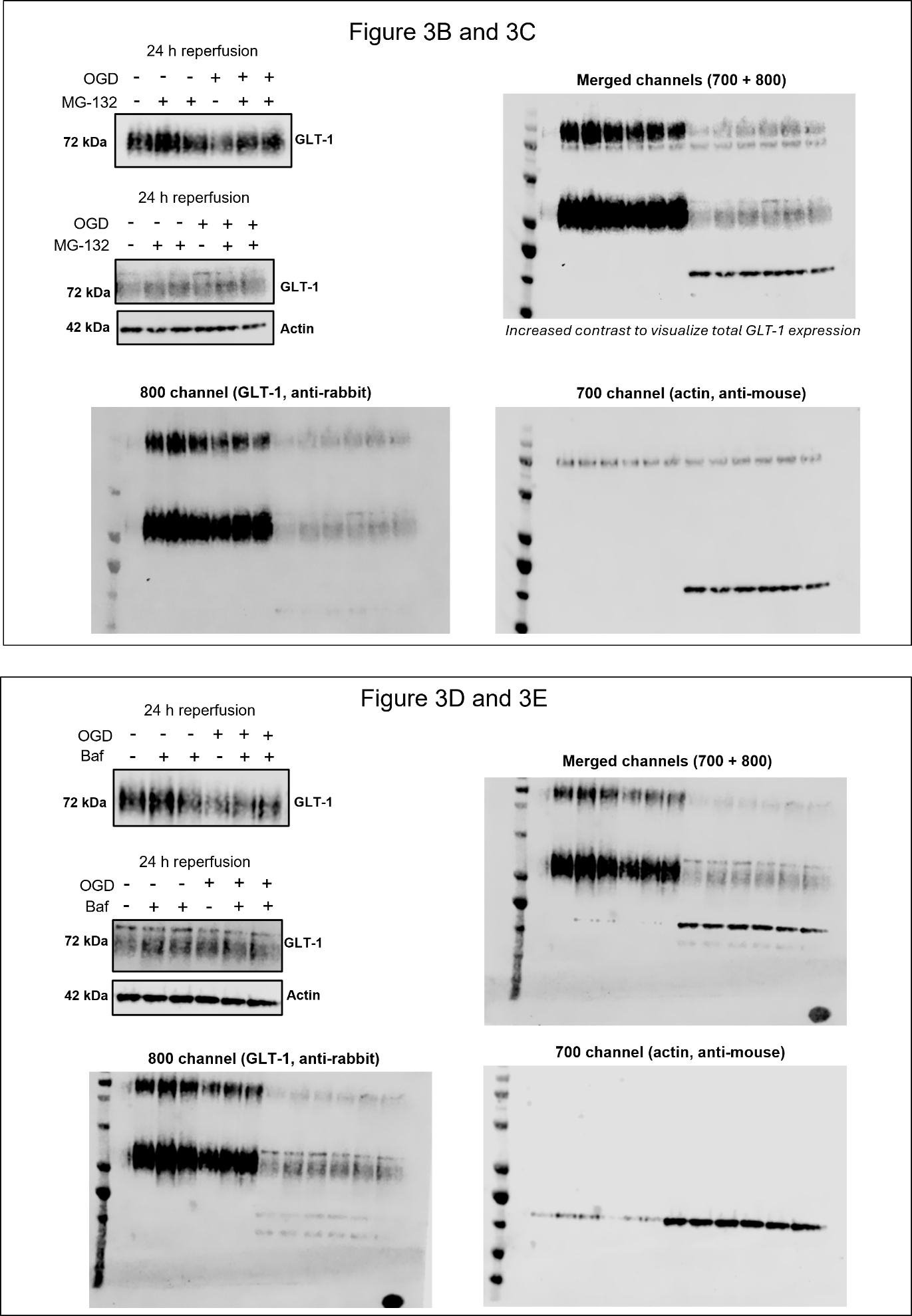
**

**
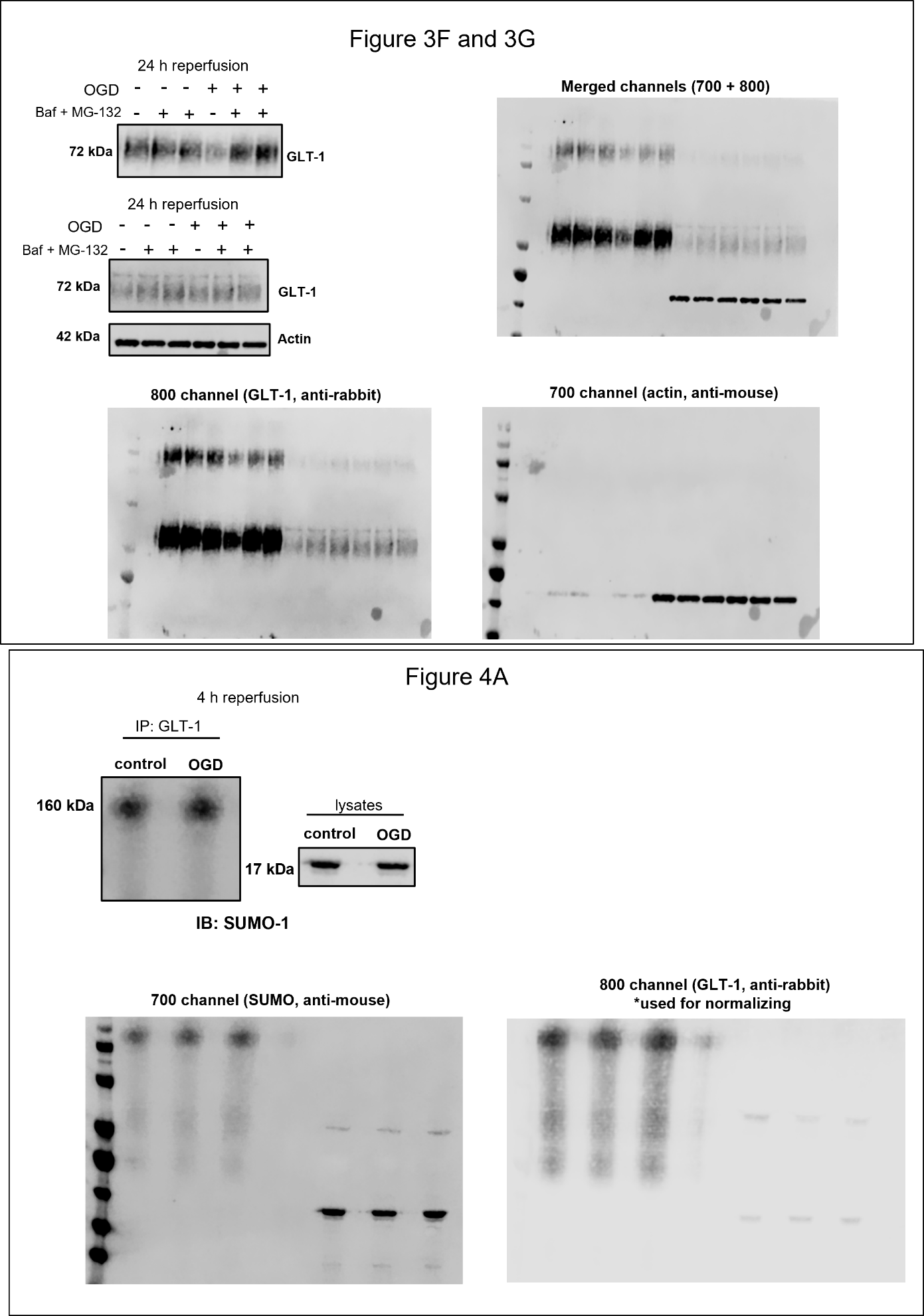

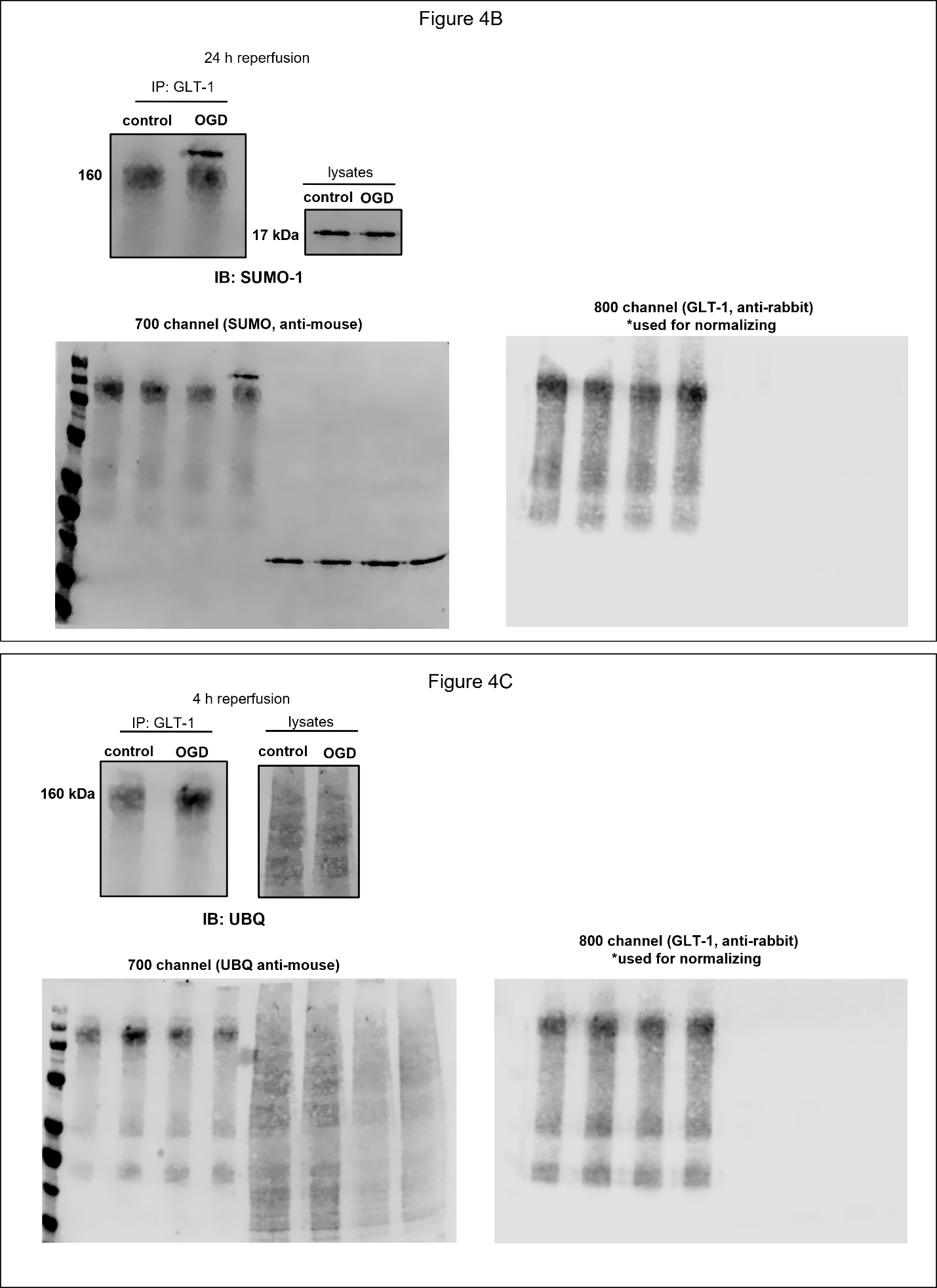

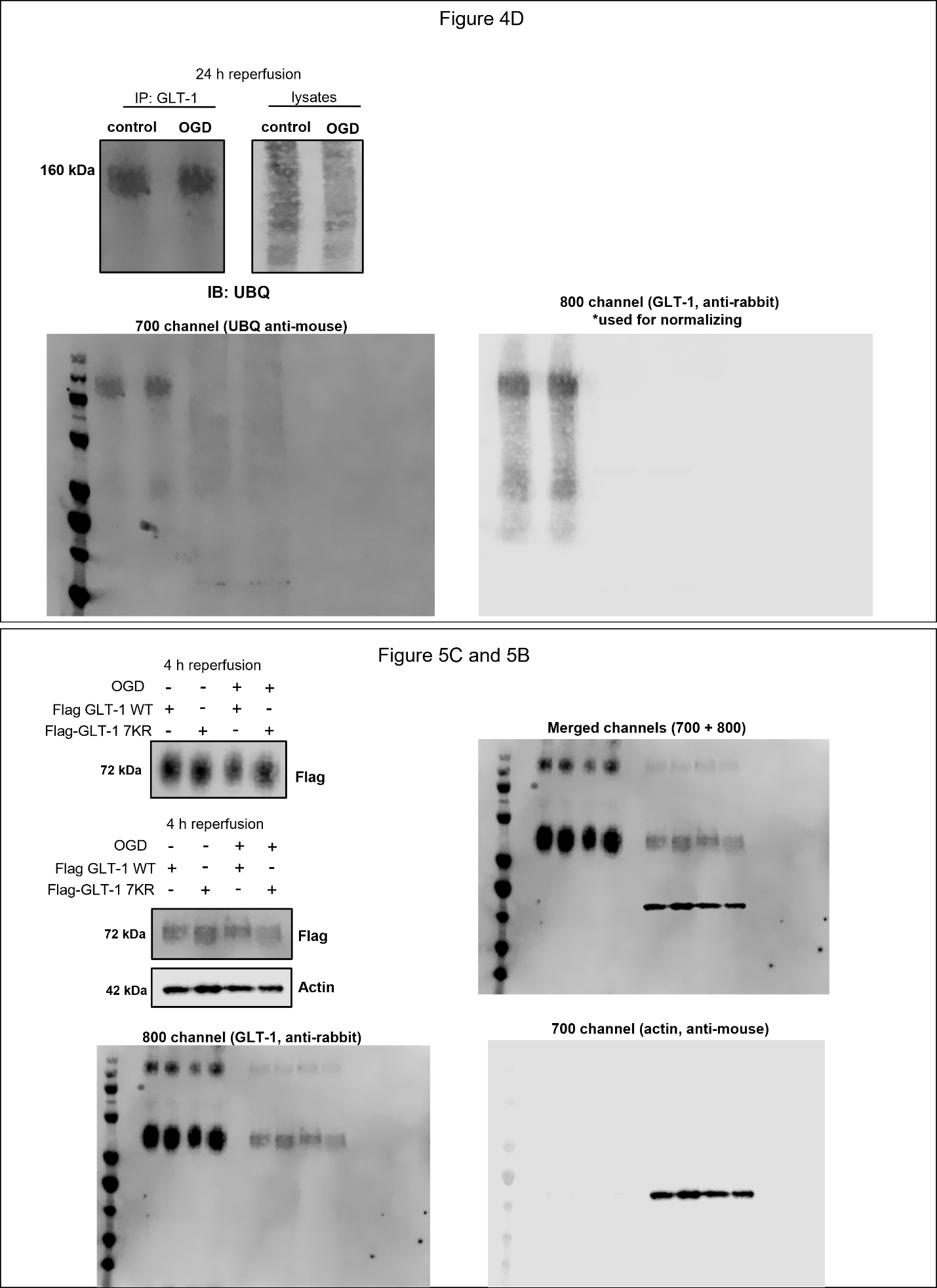

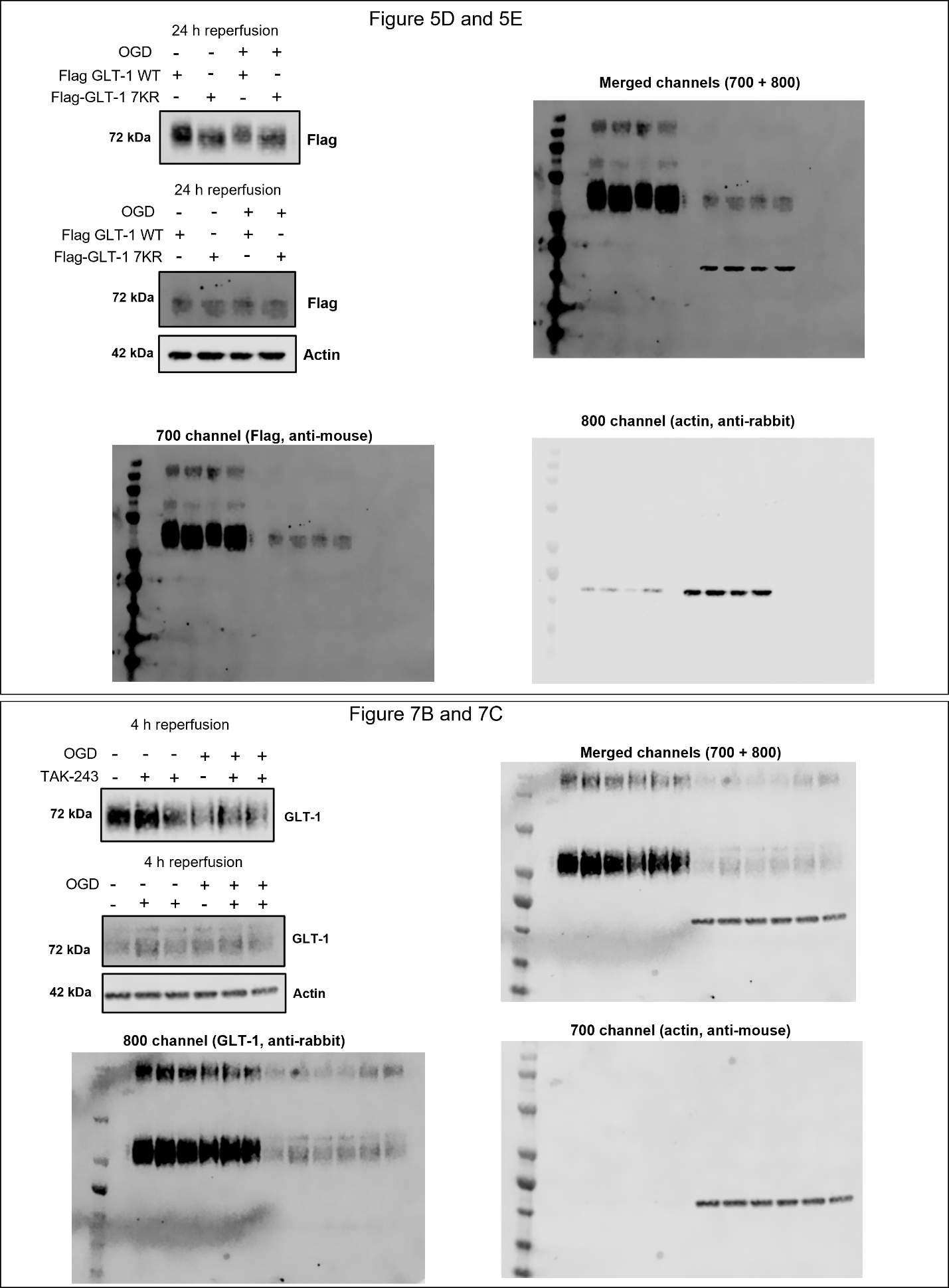

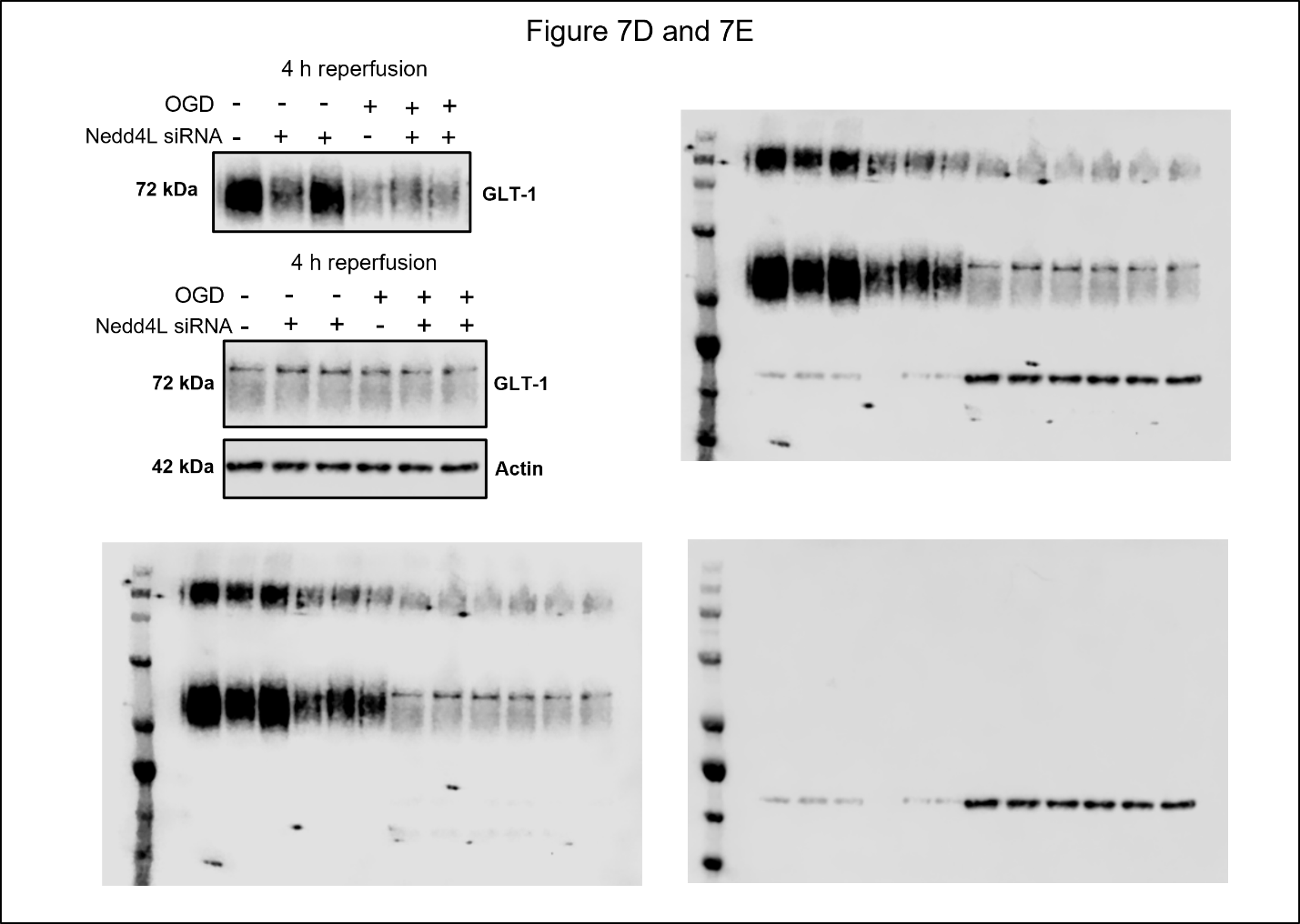
**

**
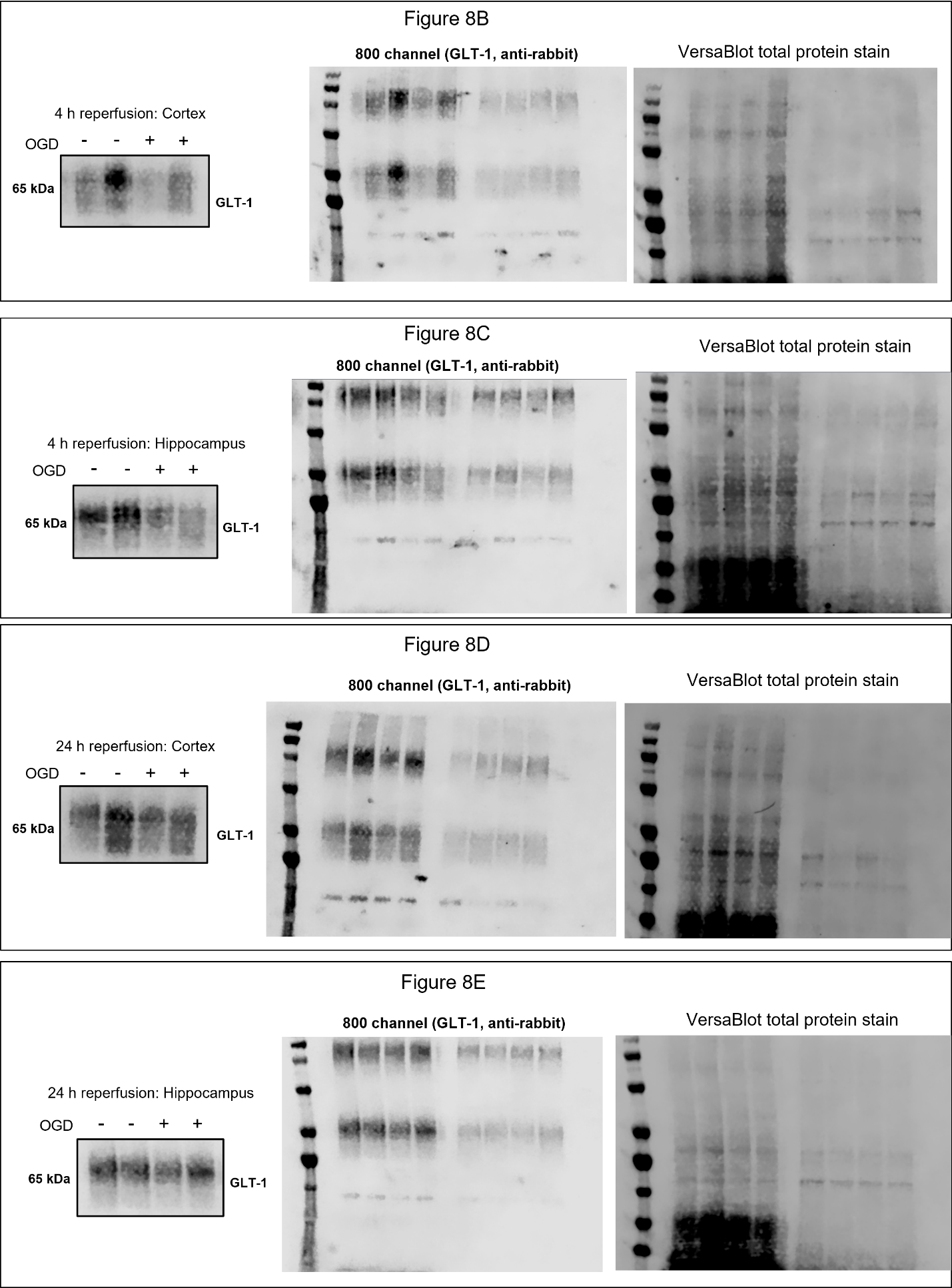
**

**
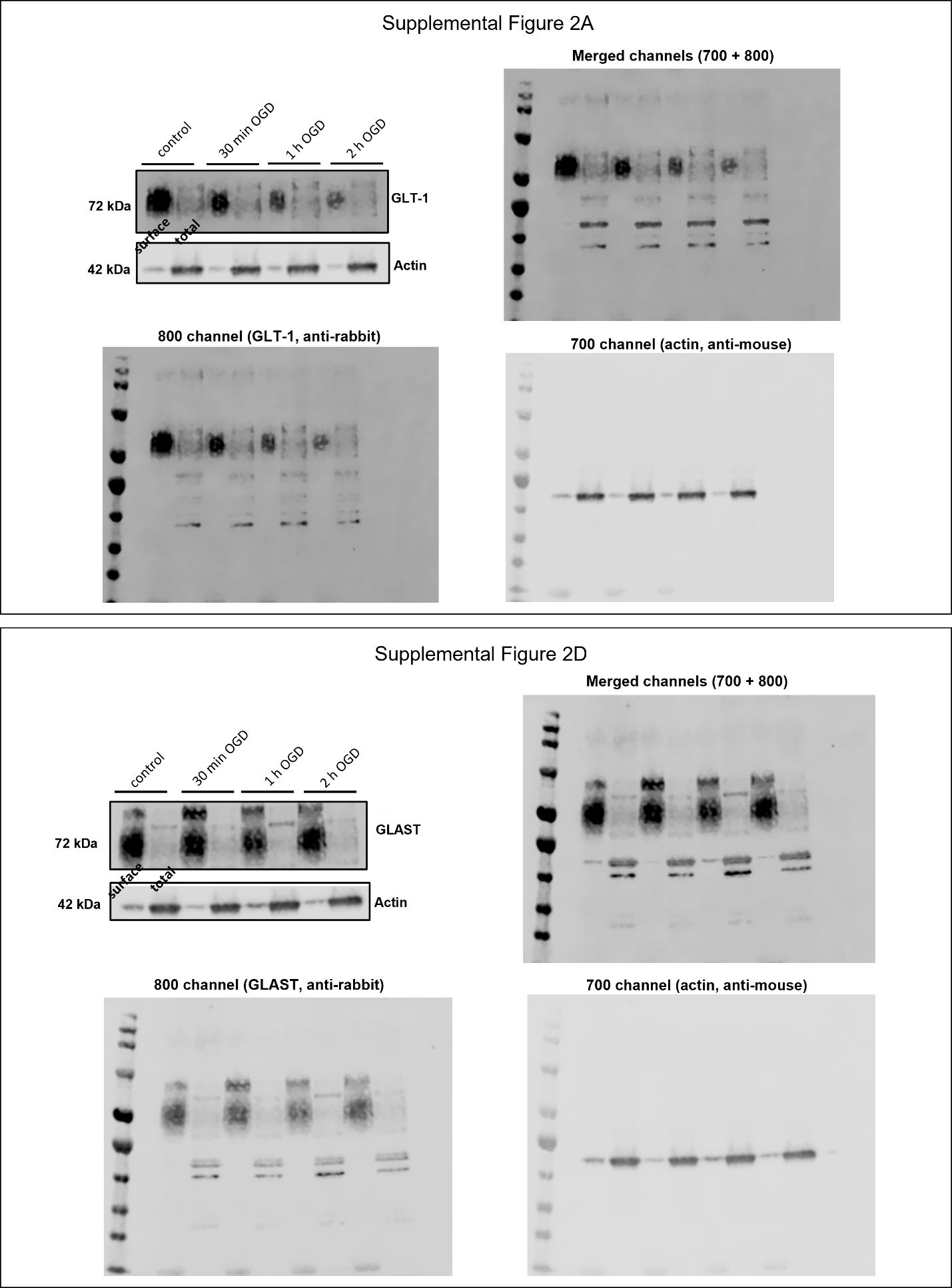
**

**
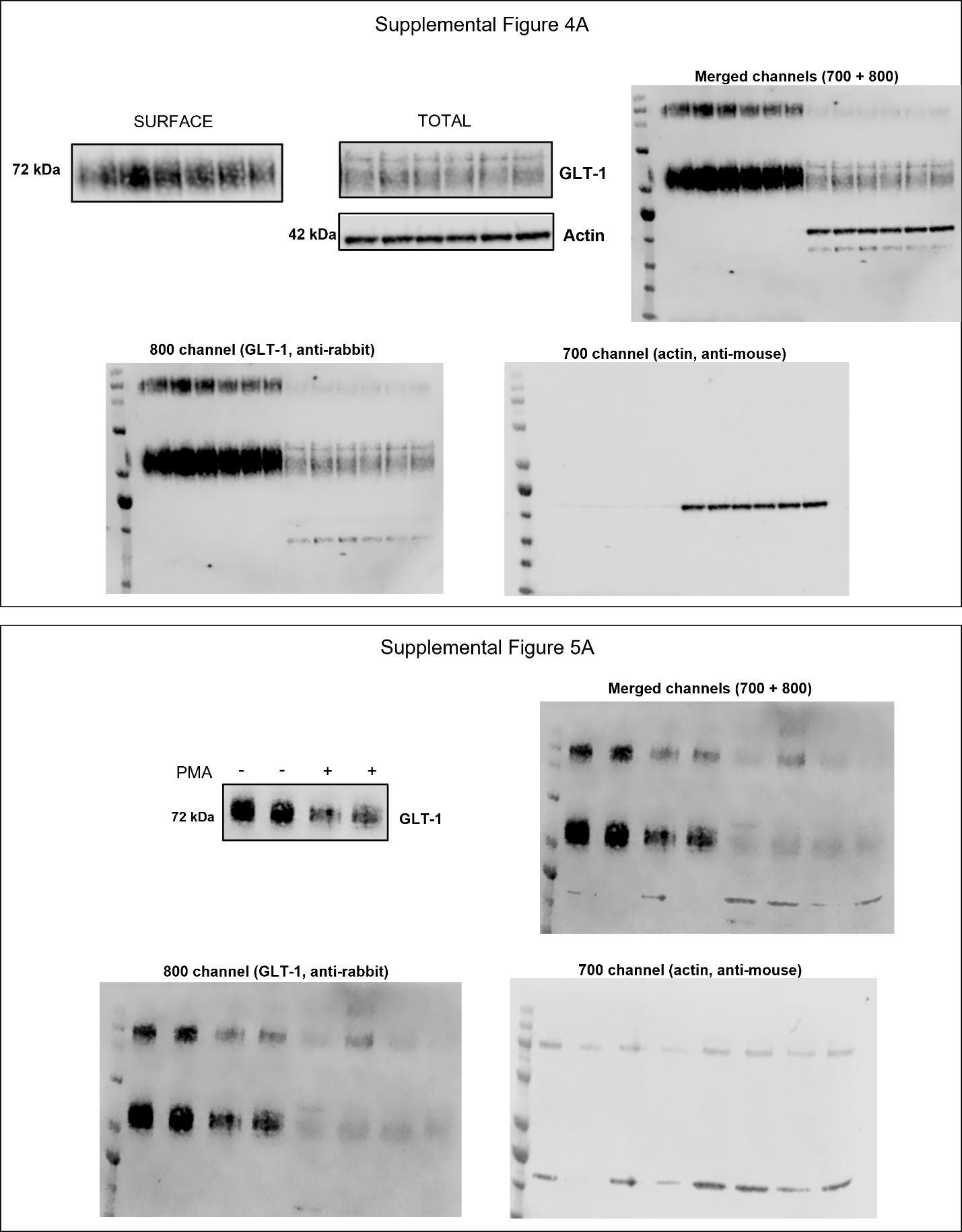
**

**
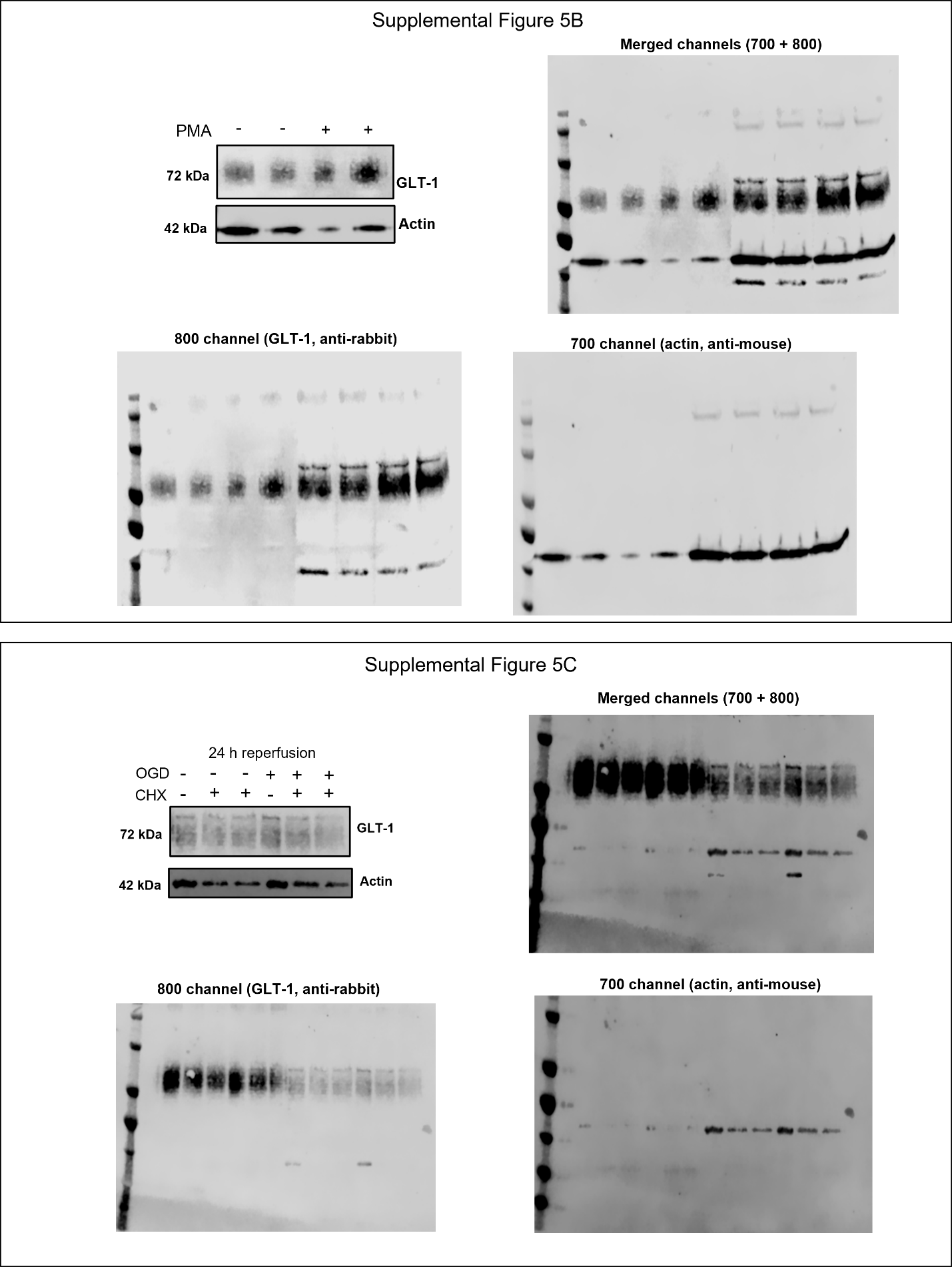
**

**
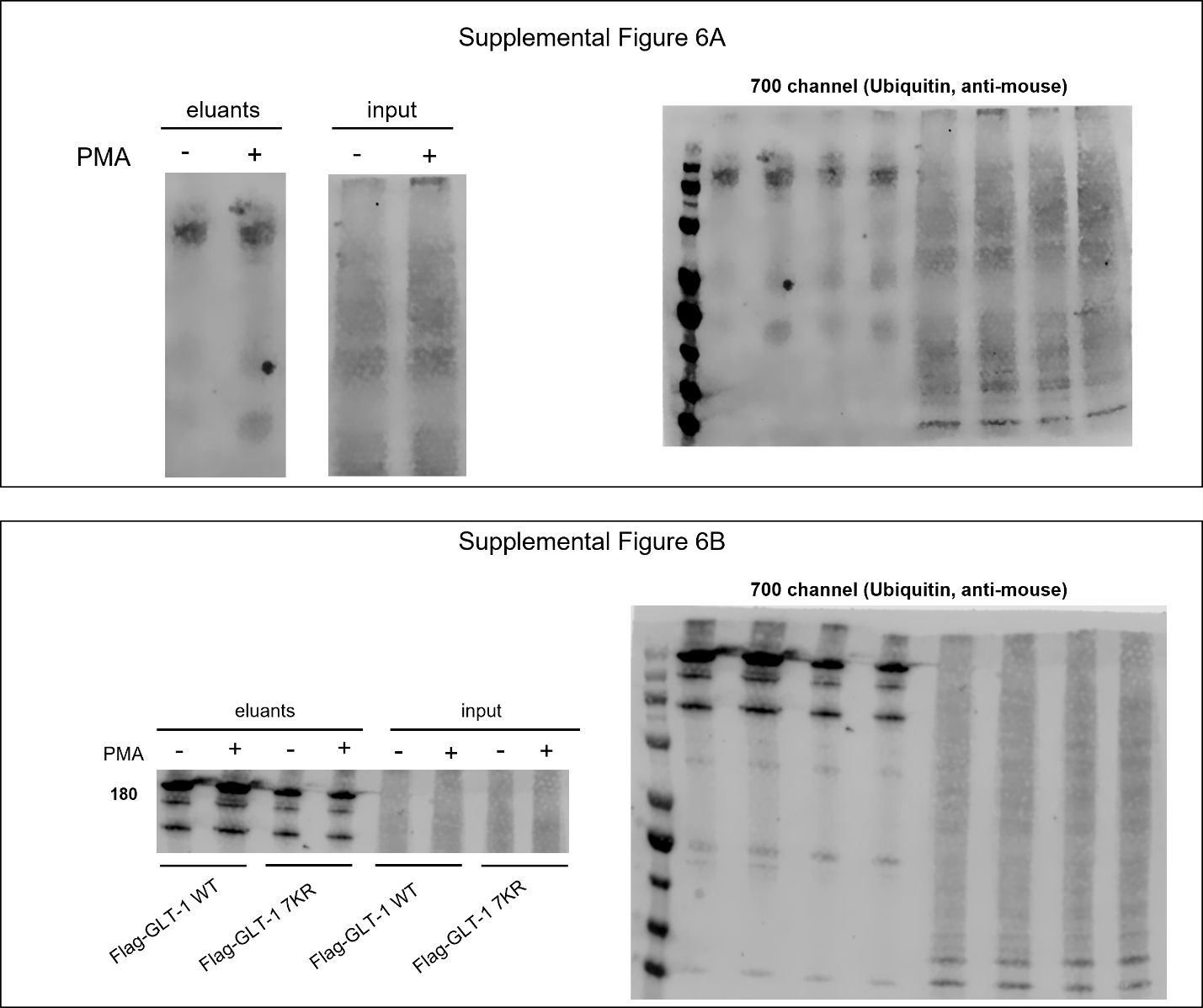
**

**
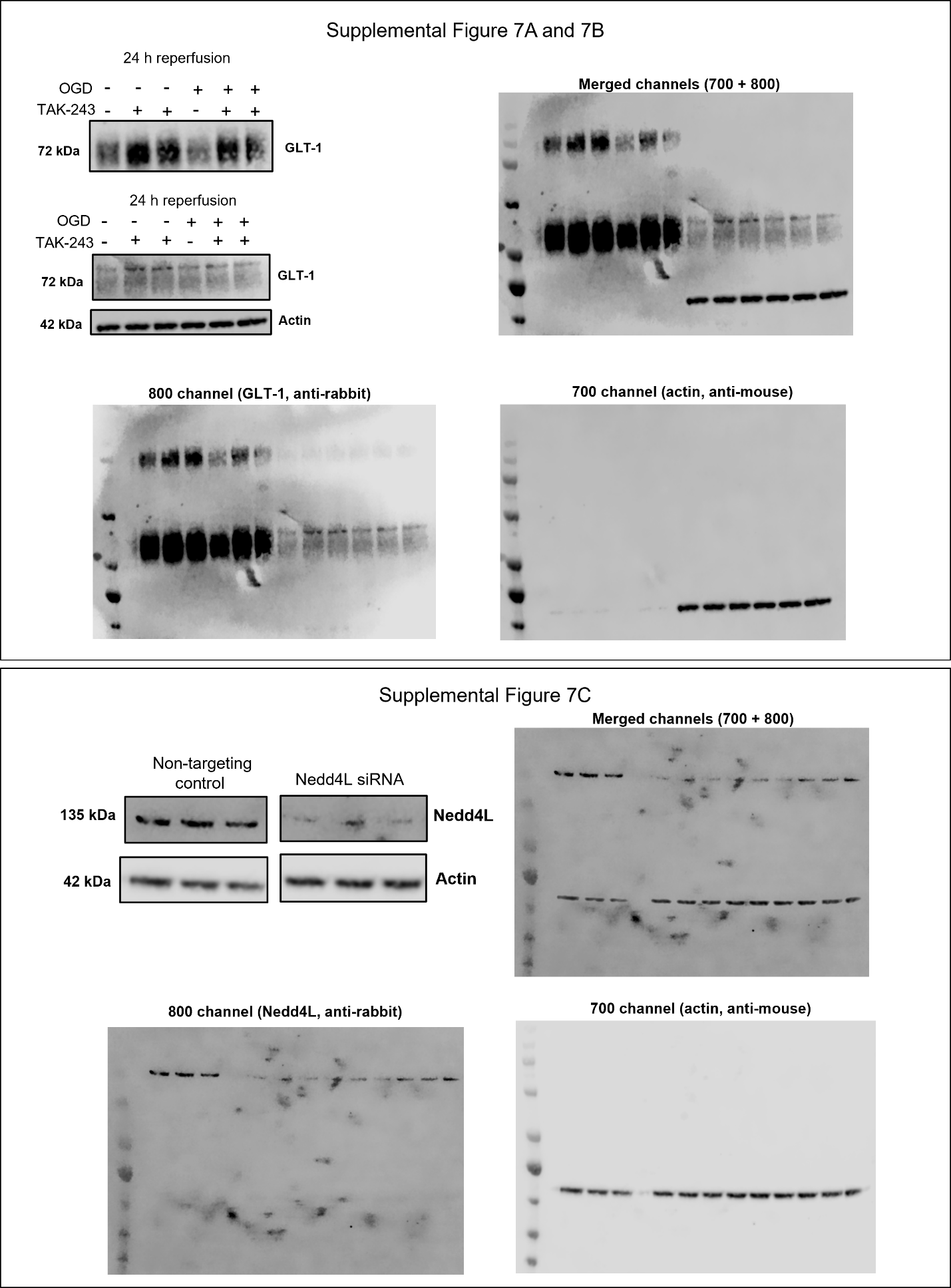
**

**
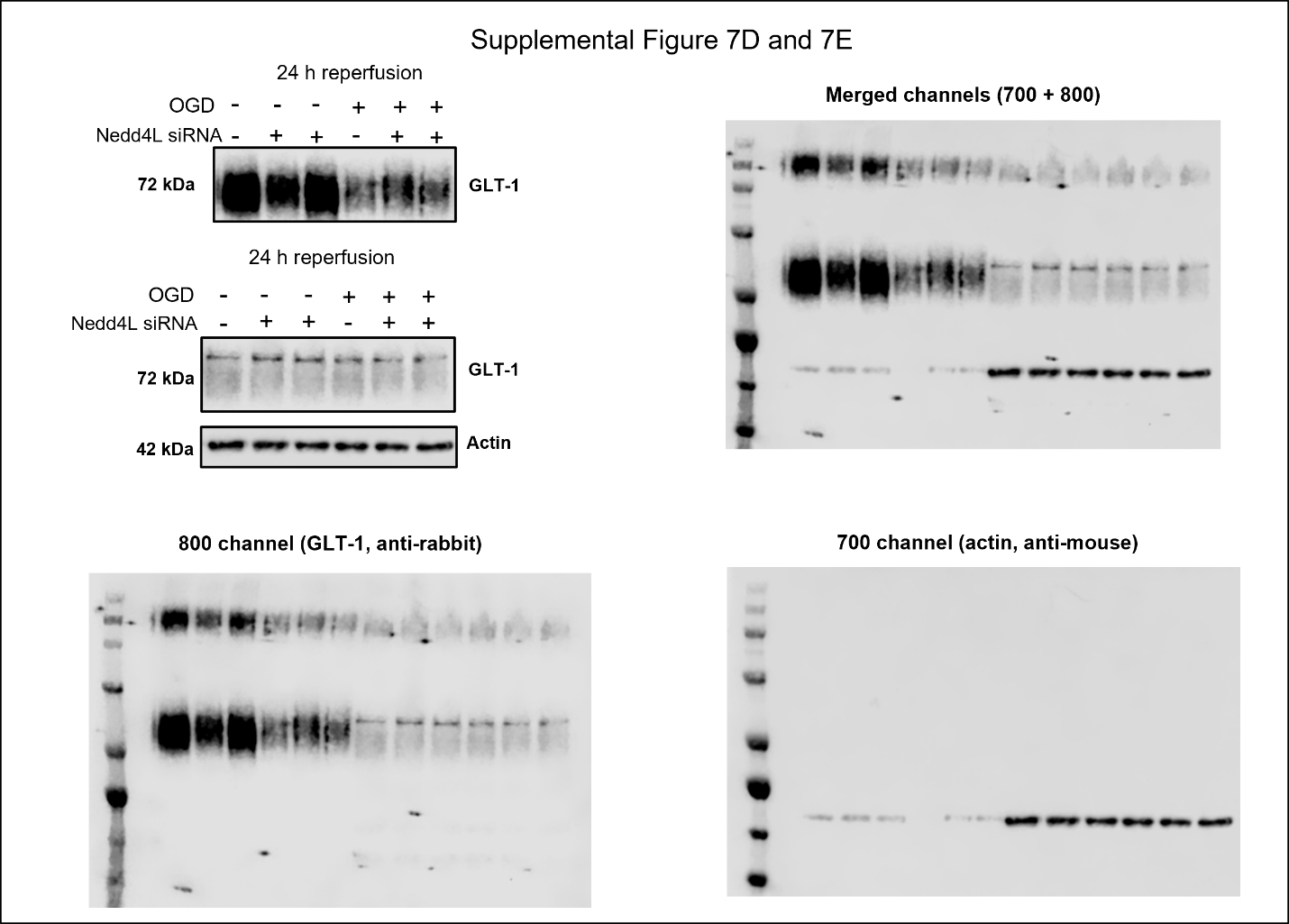
**
